# Humans Share More Preferences for Floral Phenotypes With Pollinators Than With Pests

**DOI:** 10.3389/fpls.2021.647347

**Published:** 2021-08-23

**Authors:** Victoria Ruiz-Hernández, Lize Joubert, Amador Rodríguez-Gómez, Silvia Artuso, Jonathan G. Pattrick, Perla A. Gómez, Sarah Eckerstorfer, Sarah Sophie Brandauer, Carolina G. I. Trcka-Rojas, Luis Martínez-Reina, Josh Booth, Alex Lau-Zhu, Julia Weiss, Pablo Bielza, Beverley J. Glover, Robert R. Junker, Marcos Egea-Cortines

**Affiliations:** ^1^Institute of Plant Biotechnology, Universidad Politécnica de Cartagena, Edificio I+D+I, Campus Muralla del Mar, Cartagena, Spain; ^2^Departamento de Ingeniería Agronómica, Escuela Técnica Superior de Ingenieros Agrónomos, Universidad Politécnica de Cartagena, Cartagena, Spain; ^3^Department of Plant Sciences, University of Cambridge, Cambridge, United Kingdom; ^4^Department of Biosciences, University Salzburg, Salzburg, Austria; ^5^Department of Plant Sciences, University of the Free State, Bloemfontein, South Africa; ^6^Department of Zoology, University of Oxford, Oxford, United Kingdom; ^7^Departamento de Arquitectura y Tecnología de la Edificación, Escuela Técnica Superior de Arquitectura y Edificación, Universidad Politécnica de Cartagena, Cartagena, Spain; ^8^Department of Sociology, University of Cambridge, Cambridge, United Kingdom; ^9^Oxford Institute of Clinical Psychology Training and Research, Medical Sciences Division, University of Oxford, Oxford, United Kingdom; ^10^Division of Psychiatry, Department of Brain Sciences, Imperial College London, London, United Kingdom; ^11^Evolutionary Ecology of Plants, Faculty of Biology, Philipps-University Marburg, Marburg, Germany

**Keywords:** o-acetanisole, agriculture, floral selection, humans, pest, pollinator, morphology, β-myrcene

## Abstract

Studies on the selection of floral traits usually consider pollinators and sometimes herbivores. However, humans also exert selection on floral traits of ornamental plants. We compared the preferences of bumblebees (*Bombus terrestris*), thrips (*Frankliniella occidentalis*), and humans for flowers of snapdragon. From a cross of two species, *Antirrhinum majus* and *Antirrhinum linkianum*, we selected four Recombinant Inbred Lines (RILs). We characterised scent emission from whole flowers and stamens, pollen content and viability, trichome density, floral shape, size and colour of floral parts. We tested the preferences of bumblebees, thrips, and humans for whole flowers, floral scent bouquets, stamen scent, and individual scent compounds. Humans and bumblebees showed preferences for parental species, whereas thrips preferred RILs. Colour and floral scent, in combination with other floral traits, seem relevant phenotypes for all organisms. Remarkably, visual traits override scent cues for bumblebees, although, scent is an important trait when bumblebees cannot see the flowers, and methyl benzoate was identified as a key attractant for them. The evolutionary trajectory of flowers is the result of multiple floral traits interacting with different organisms with different habits and modes of interaction.

## Introduction

The floral phenotype is shaped by diverse selective forces including pollinators and pests, and also – at least in the case of ornamental flowers – humans (Gessert, 1993; Ågren, 2019; Ramos and Schiestl, 2019). To maximise fitness, plants need to find a balance between attracting pollinators and repelling antagonists (Junker and Blüthgen, 2010). Thus, both pollinators and herbivores exert pressures on the selection of floral traits, driving the evolution of angiosperms in natural ecosystems. However, the trade-off between attraction and defence is also important in agricultural systems and thus is of economic relevance.

Humans have long selected flowering plants (8,000–10,000years) not only as a food resource, but also for their ornamental attributes (Milla et al., 2015). There is evidence of the study of ornamental plants dating from C.E. 1,090 (Stoskopf et al., 1994) and wherever humans live, the use of plants as ornaments is ubiquitous in their urban and periurban areas (Kowarik, 2005). The domestication of wild plants through selective breeding is the basis of agriculture, and it allows wild plants to be transformed into economically desirable crops (Stoskopf et al., 1994). Nonetheless, artificial selection, and thus domestication, is constrained by the interaction of plant genotypes with biotic and abiotic factors in the environment, as well as by biophysical, physiological, developmental, and genetic factors (Milla et al., 2015). For instance, in the case of ornamental flowering plants, which are presumably selected by humans, biotic selection of the floral phenotype is affected by the perception and preferences of the tripartite forces of humans, florivores, and pollinators.

Humans respond to and interact with their environment based on a multimodal comprehension of it (Storms and Zyda, 2000; Lindemann-Matthies et al., 2010). Although, ornamental plants have been studied from several perspectives (Reichard and White, 2001; Kingsley et al., 2009; Kendal et al., 2012; Erickson et al., 2019), little is known about the specific floral traits that might be more relevant for humans when they choose which flowers to grow (Rahnema et al., 2019). Studies on the preferences of plant breeders can provide information about the most important floral attributes guiding their decision-making based on aesthetic values. On the other hand, bumblebees (*Bombus* spp.) are model organisms for studying pollination in an ecological and evolutionary context (Wilmsen et al., 2017). Numerous studies have revealed that multiple floral phenotypes are perceived by bumblebees and affect their behaviour when selecting which flowers to visit (Katzenberger et al., 2013; Whitney et al., 2013; Bailes et al., 2018). Furthermore, they are also of major importance in agriculture, pollinating crops and increasing yield quantity and quality (Bailes et al., 2018). In contrast, although, thrips are considered secondary pollinators (Terry, 2002), they are more widely known for causing damage to plants. Their perception and selection of flowers is known to be influenced by multiple plant stimuli (Cao et al., 2018). In the context of agriculture, the western flower thrip, *Frankliniella occidentalis*, is a globally dispersed pest with a wide plant host range (Reitz, 2009; He et al., 2020). The interaction of these three organisms with plants is of great economic relevance, since they may synchronously exert selective pressures on the floral phenotype.

It has been demonstrated that both mutualistic bumblebees and antagonistic visitors exert selective pressures on floral phenotypes (Ramos and Schiestl, 2019). However, whether humans reinforce the selection by mutualists or antagonists, or exert opposing selective pressures on the floral phenotype, has been overlooked. Studies integrating behavioural responses of insects and humans to plants can reveal the traits under selection by each of these organisms. Since responses of humans, bumblebees, and thrips to flowers are the result of multimodal decisions (Storms and Zyda, 2000; Katzenberger et al., 2013; Terry et al., 2014; Telles et al., 2017; Wilmsen et al., 2017), holistic approaches are required to pinpoint, which are the most important floral traits underlying the floral selection of these organisms. In addition, behavioural experiments are needed to assess single traits that may be under selection (Junker and Parachnowitsch, 2015).

An ideal model for studying floral traits under pressure for selection is *Antirrhinum*. The genus was described by Plinius as a classical Roman ornamental (Stubbe, 1966). *Antirrhinum* has been long studied for its interest from ecological and evolutionary standpoints (Glover and Martin, 1998; Schwarz-Sommer et al., 2003), but also for its use in agriculture in the emerging market of edible-flowers (Rop et al., 2012; González-Barrio et al., 2018; Stefaniak and Grzeszczuk, 2019) and its historical and economic value as an ornamental (Kowarik, 2005). In this study, we assessed the preferences of *Bombus terrestris*, human plant experts and *F. occidentalis* for different flowers of *Antirrhinum* sp., including four Recombinant Inbred Lines (RILs) displaying a range of floral phenotypes. We phenotyped flowers with respect to colour, morphology, sizes of different floral parts, composition and quantity of scent emission in stamens and flowers, pollen content and viability, and trichome density. Our aim was to compare the preferences of pollinating bumblebees, antagonistic thrips, and humans for *Antirrhinum* flowers and to identify the flower traits potentially under selection by these organisms. These findings may be helpful for the understanding of evolutionary trajectories and also may inform agricultural practices.

## Materials and Methods

### Flowers

**Figure 1:**
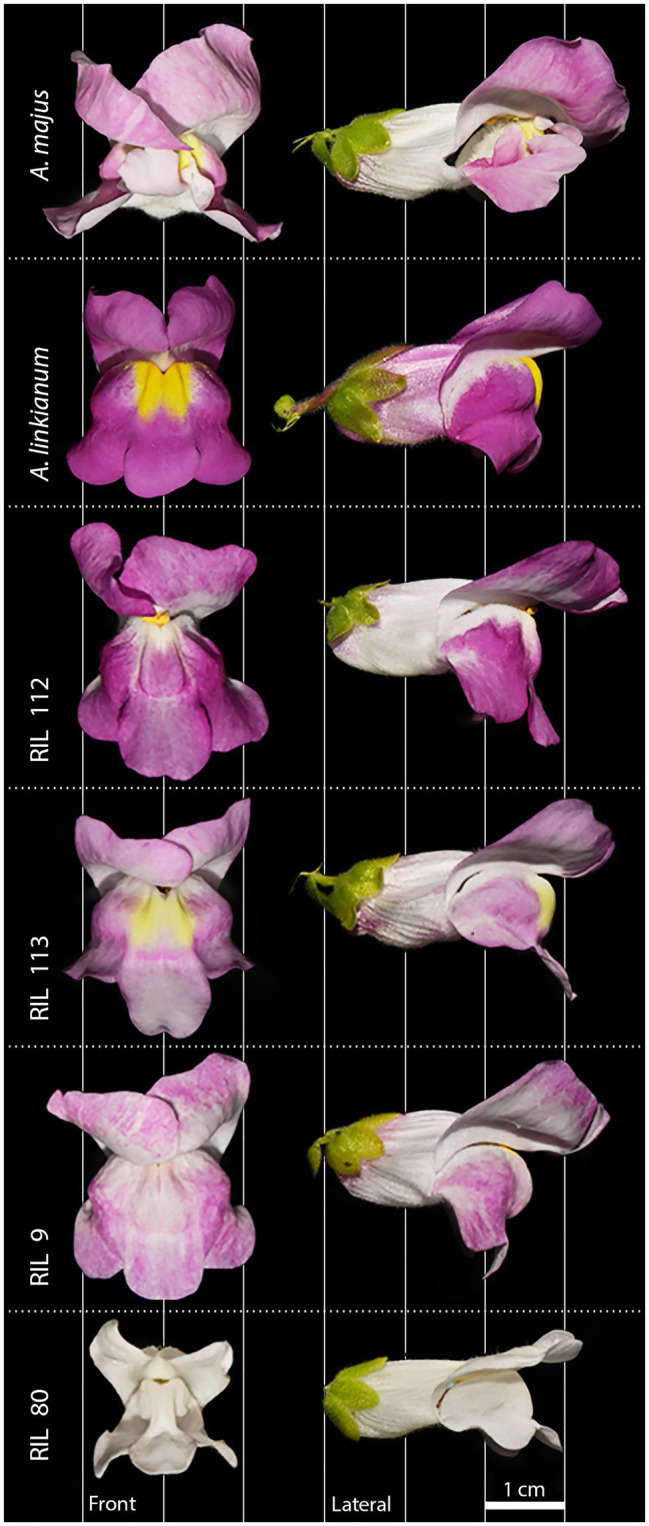
Flowers from lines used for experiments. Parental species: *Antirrhinum majus* and *Antirrhinum linkianum*. Recombinant Inbred Lines (RILs): 112, 113, 9, and 80. Front and lateral view.

We performed a cross between *Antirrhinum majus* line 165E, a line deriving from the John Innes JI75 line (Schwarz-Sommer et al., 2003), and *Antirrhinum linkianum* (Botanic Garden, University of Coimbra, Portugal; Ruiz-Hernández et al., 2017). A single F1 plant was used to obtain an F2. The recombinant inbred line was further developed in a standard way i.e., plants were selfed and maintained by single-seed descent. We selected four RILs, with contrasting scent profiles, on F5–F7 populations: these were identified as lines 9, 80, 112, and 113. We used fully developed flowers, at their maximum stage of scent emission (3–4days after anthesis; Weiss et al., 2016) for all analysis and experiments (Figure 1).

### Plant Phenotyping

#### Colour of Floral Parts

**Figure 2:**
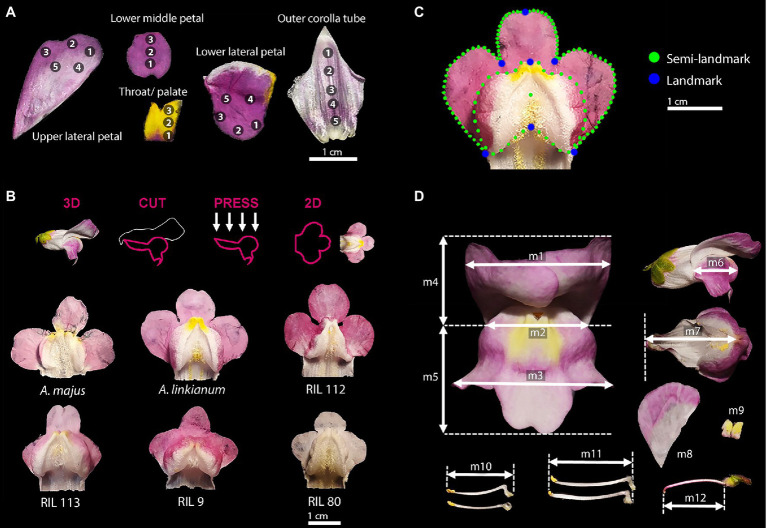
Floral parts phenotyped. **(A)** Floral parts used to measure the colour of flowers. Numbers indicate the location of measurements on each floral part. **(B)** Dorsal and lateral petal lobe dissection used to analyse floral morphology. **(C)** Landmarks and semilandmarks used to analyse floral morphology. **(D)** Location of floral size measurements for each flower. Length of front (M1–5), lateral (M6), lower lobe (M7), short and long stamen (M10 and M11), and gynoecium (M12). Area of upper lateral petal (M8), throat/palate (M9).

We dissected the flowers, flattened them and glued each petal section to black, non-reflective cardboard to prevent light scattering from curved petal surfaces. Each mounted petal was analysed, whilst placed in a box lined with black cardboard and illuminated with a Deuterium-Halogen light source (Ocean Optics DH 2000). The background material was corrected for and the reflectance spectrum of each petal section was measured, relative to a white standard, with an Ocean Optics USB2000+ Spectrometer at an integration time of 10ms. The petals were flattened to reduce any artefact caused by light scattering-off of curved petal surfaces. Reflectance spectra were analysed with SpectraSuite Version 1.0 (Ocean Optics). We measured five floral parts: upper and lower lateral petal, lower middle petal, palate, and outer corolla tube (Figure 2A). Flower colour was measured in 21 different spots, with 15–19 replicates per line. Colour spectral data was processed using the R package *pavo* (Maia et al., 2019), and measurements were restricted to the range of wavelengths visible to insects and humans (300–700nm). Flowers of the genus *Antirrhinum* are usually multicoloured and the multiple measurements on each flower resulted in *n*=21 different reflectance spectra. In order to best represent colour differences between flowers, we calculated the Euclidean distances between the reflectance spectra per spot, which means the colour of the same petal position was compared between two flowers. This procedure resulted in *n*=21 Euclidean distances per pair of flowers, the mean value of these distances was defined as the average colour distance between two flowers.

#### Morphology

We dissected the flowers by cutting along the tube with a razor blade, in correspondence with the hinge of the dorsal and lateral petal lobes (Figure 2B). As a proxy for floral morphology, we estimated the shape of the ventral petal (Figure 2C). Following (Cui et al., 2010), we flattened the dissected three-lobed lips by gluing them onto black paper and photographed them in a standardised manner. The software tpsDig2 2.31 and tpsUtil 1.76 (Rohlf, 2015) was used to digitize 139 landmark and semilandmark points (Figure 2C). Using the R-package GEOMORPH 3.1.0 (Adams and Otárola-Castillo, 2013), landmark coordinates were subjected to a Generalised Procrustes Superimposition with semilandmarks slided based on minimising bending energy. The effect of asymmetry was removed by extracting the symmetric component of the shape (Klingenberg et al., 2002), using *bilat.symmetry* from GEOMORPH.

#### Size of Floral Parts

We used the image processing package Fiji (Schindelin et al., 2012) to measure the sizes of complete and dissected flowers. We took scaled pictures of 9–17 replicates per line. We measured the dimensions of 10 floral parts: flower front (M1–5), flower lateral (M6), lower petal lobe (M7), short and long stamen (M10 and M11), and gynoecium (M12); and the area of the upper lateral petal lobes (M8) and palate of each flower (M9; Figure 2D).

#### Volatile Organic Compound Analysis

Scent emission was analysed using GC-MS and Twisters™ (Gerstel, Mülheim an der Ruhr, Germany) as detailed in Ruiz-Hernández et al. (2018). Cut flowers were placed inside glass beakers and cut stamens were placed inside 10ml headspace vials (Supplementary Figure S1). Beakers and vials were positioned in 2L desiccators for 24h inside a growth chamber under a regime of 16:8h light-dark and 23–18°C conditions. A minimum of 10 replicates (in the case of RILs: five from F5 and five from F7) from different plants within each line were analysed by GC-MS.

Scent profiles were determined using the R package gcProfileMakeR (Pérez-Sanz et al., 2020). Compounds present in at least 90% of the replicates, with a minimum average quality of 90%, were selected as representatives of the scent profile of each line. Linear retention indexes (LRI) were calculated for tentatively identifying compounds by comparing available LRIs in the literature. For that purpose, we used the retention times (RT) of C8–C20 alkanes (Sigma Aldrich, 04070), analysed under the same chromatographic conditions as flower samples (Zellner et al., 2008; Ruiz-Hernández et al., 2018; Supplementary Table S1). We used acetophenone, methyl benzoate, methyl cinnamate, methyl salycilate, and ocimene, (Sigma-Aldrich, 42,163, 18,344, 96,410, 240,826 and W353901, respectively) as standards for chromatographic identification. Average total scent emission (mg) was calculated by using an external calibration curve obtained by adding standards to the sampling system (y=5.247*10^8^*x; Ruiz-Hernández et al., 2018; Table 1). Multivariate analysis and Random Forest analysis were performed using the total peak area divided by the fresh weight of samples (Ruiz-Hernández et al., 2018). Due to the difficulties found when exactly quantifying the emission of single volatile organic compounds (VOCs) in complex matrices (Ruiz-Hernández et al., 2018), we decided to use a general dilution of 1:1,000 of VOCs in bioassays with thrips and bumblebees. Differing dosages were chosen to test hypotheses generated from preference tests and Random Forest analyses. Synthetic standards used in bioassays with thrips and bumblebees were: α-farnesene (W383902, Sigma-Aldrich, mix of isomers), methyl benzoate (M29908 Sigma-Aldrich, 99%), α-acetanisole, (M9203 Sigma Aldrich, 99%), cinnamyl alcohol (standard provided by Prof. em. Manfred Kaib, University Bayreuth, Germany), methyl cinnamate (standard provided by Günther Gerlach, SNBS Munich, Germany), and β-myrcene (EGA-Chemie M10,000-5, technical quality).

**Table 1:** Mean percentage of VOCs in scent profiles of *A. majus*, *A. linkianum*, and RILs 112, 113, 80, and 9.

VOC	*Antirrhinum majus* *n*=10	*Antirrhinum linkianum* *n*=10	RIL 112 *n*=13	RIL 113 *n*=10	RIL 9 *n*=10	RIL 80 *n*=10
o-acetanisole	0.4					
**Acetophenone**	17.8	3.1	8.5	44.2	4.5	41.1
o-acetylphenol	0.6		1.3	5.1	0.8	
Benzenepropanol			3.7		0.3	
(E)-cinnamaldehyde					0.9	
Cinnamyl alcohol			7.1	2.1	11.4	8.6
Decanal	0.2			0.3		0.3
3,5-dimethoxytoluene						2.3
Ethyl benzoate	0.7		0.9	0.3		
Eremophilene		3.5			8.4	
α-farnesene	1.3	20.4	11.7	14.1	11.2	15.7
Hexahydrofarnesyl acetone			0.5		0.2	0.3
Linalool					1.6	
**Methyl benzoate**	33		22.4	11.0	7.6	11.0
**Methyl cinnamate**		6.8	8.5	3.0	2.5	2.5
Methyl hydrocinnamate					3.8	0.3
Methyl 2-methyl butyrate				0.2		
**Methyl salicylate**				4.9	4.1	4.2
β-myrcene	7.4	6.4	5.0	0.8	5.7	3.2
Nonanal				0.4		0.3
**(E)-ocimene**	38.2	57.7	29.5	12.2	36.7	8.1
Sabinene		1.0		0.2		
**St. Acetophenone**				1.0		0.8
St. Hexahydrofarnesyl acetone		0.9	0.7	0.3	0.2	
St. α-farnesene						0.4
**St. Methyl benzoate**	0.3				0.1	0.6
St. Nonanal						0.1
St. β-pinene		0.3				
Average total scent emission (mg)	3.074±1.125 ab	1.847±0.879 bc	1.394±0.709 c	2.669±1.097 abc	3.804±1.811 a	3.155±0.680 ab

Volatile organic compounds obtained from stamens are indicated with “St.”. VOCs in bold were identified with authentic standards and linear retention indexes (LRIs), the rest were tentatively identified with LRIs. Last row shows the average total emission of each line (mg) and its SD. Different letters indicate statistical differences between lines, according to Tukey test.

#### Pollen Viability and Content

To determine the percentage of viable pollen grains in each flower, the fluorescein diacetate staining method was used (Heslop-Harrison and Heslop-Harrison, 1970; Li, 2011). Flowers were inspected daily and all the stamens were removed from a flower on the day of dehiscence. Stamens were placed in 1.5ml tubes with 250μl of fluorecein diacetate in BK-Buffer. Tubes were vortexed for 60s and stamens were removed from tubes and visually inspected to ensure that all pollen had been released from the anthers. Each tube was briefly vortexed again to ensure even distribution of pollen grains and a 20μl subsample of the pollen suspension was immediately pipetted into each of the two 9mm^2^ grids with a depth of 0.2mm of a Modified Fuchs Rosenthal Chamber (MFS; Rohem, India). Pollen samples were illuminated using a CoolLED pE300 White Fluorescence illumination system and imaged using a Nikon Eclipse 50i microscope with a 40X objective and a mounted GT Vision GXCAM HiChrome-S tablet. Viable, fluorescent pollen and non-viable, non-fluorescent pollen (Supplementary Figure S2) was counted separately to determine the percentage of viable pollen using CountThings from Photos (Dynamic Ventures, Inc. d/b/a CountThings). The total amount of pollen was calculated adding viable and non-viable pollen. The number of flowers phenotyped per line was 8–14. At least three different 1mm^2^ chambers were counted for each flower.

Total pollen content of each flower was calculated by using the average pollen count for the sample (*n*) divided by the volume of the grid of the Modified Fuchs Rosenthal Chamber on which the pollen grains were counted (1.8μl) and multiplied by the volume of the fluorecein diacetate solution in which the pollen was suspended (250μl):

$$Totalnumberofpollengrains\;per\;flower=250\frac{n}{1.8}$$

#### Density of Trichomes

Using a scanning electron microscope (HITACHI S-3500N), we counted the number of trichomes on the palate of flowers. We had three replicates per line and we dissected 5mm^2^ of each flower. We took three images in different areas of each replicate (scale: 200, magnification: x180, size: 910×683μm). We followed a protocol for critical point drying with glutaraldehyde, ethanol, and acetone (Manchado-Rojo et al., 2014). Due to the length of trichomes, we cut them using tape and tweezers (Supplementary Figures S3A–D). We counted the number of trichome bases as a proxy for trichome number in the area examined, giving trichome density.

### Experimental Design With Flowers

We tested preferences of bumblebees, humans, and thrips for flowers of the different *Antirrhinum* lines studied. Preferences were assessed pairwise, contrasting parental line *A. majus* against *A. linkianum*, RIL 112, RIL 113, RIL 9, and RIL 80. In the case of bumblebees and humans, two types of experiments were performed: (1) Using whole flowers and (2) Using floral scent in isolation. In contrast, assays with thrips were performed after first separating flowers from stamens and testing flowers without stamens and stamens, independently.

Whilst bumblebees are well known for pollinating *Antirrhinum* plants (Jaworski et al., 2015) and humans have used this genus long as ornamental (Kowarik, 2005), the antagonistic effect of thrips on these plants may be either caused by a strong pollen reduction or by the transmission of pathogens (Ullman et al., 2002).

#### Bumblebee Experiments With Flowers

For experiments with flowers, *B. terrestris audax* colonies were obtained from Biobest Group NV (Westerlo, Belgium), supplied by Agralan (United Kingdom), and connected by a transparent tube to the flight arena, a 0.3×0.75×1.12m plywood box with a clear UV- transparent Plexiglass lid (Bailes et al., 2018). Colonies were fed *ad libitum* with ~30% w/v sucrose solution, which was also used as a reward in experiments. At least 10 bumblebees performed each pairwise-experiment, but each bee was tested independently in the flight arena. Bumblebees were pre-trained to feed from 13cm tall feeding towers composed of black card wrapped with black tape sitting within “Aracon” bases (Lehle, Roundrock, TX). Towers were covered with plastic mesh supporting a microcentrifuge tube lid (Supplementary Figure S4A) containing sucrose solution. Tower-feeding foraging worker bumblebees were marked on the thorax with water-soluble paints and used for further experiments. Some bumblebees were used more than once and, in those cases, at least 7days were left between assays to allow short term learning-associations to disappear from their memories (Jaworski et al., 2015). Consequently, we consider our results in the context of flower naive responses of bumblebees, here testing their innate preferences to the different floral traits. Scent experiments were carried out by hiding flowers inside cardboard towers (1:1). Supplementary Figures S4A,B illustrate how flowers were displayed for experiments with floral scent alone and with whole flowers, respectively. Flowers were kept in contact with cotton dampened in a 5% w/v sucrose solution to keep the turgor pressure. Each time a bee fed from a tower or a flower, sugar solution was refilled and distribution of towers/flowers was changed.

#### Bumblebee Preference Assessments

To test the innate preferences of bumblebees for whole flowers, we displayed five flowers of each pair pseudo-randomly in the arena and let them choose to feed on them 10 times. Inside each flower, we placed a cut yellow micropipette tip supplied with 20μl of sucrose solution to ensure equal availability of reward (Supplementary Figures S4C,D). We counted each time bumblebees fed from a flower-tip as a positive choice, refilled the tip and changed the layout of flowers in the arena. Some flowers were used for testing bumblebees more than once. In these cases, flowers were not used for at least 2h between experiments. Bumblebees deposit cuticular hydrocarbons, whilst visiting flowers, and can subsequently use these “scent marks” as a cue, influencing their floral choices (Goulson, 2010). Although, the cues themselves may last for over 24h (Witjes and Eltz, 2009), *B. terrestris* appear to stop using the cues by the time 1h has passed since the flower was previously visited (Stout et al., 1998). Bumblebees may be able to learn to use scent marks that are older than 1h (Goulson, 2010); however, here, we were examining innate rather than learned preferences, and so leaving the flowers for 2h should avoid any potential influence of scent markings.

For floral scent experiments, 10 towers, five with a flower of each line from the pair being tested, were distributed pseudo-randomly in the flight arena. Microcentrifuge tube lids were supplied with 20μl of sucrose solution. Each bee was allowed to feed from towers 10 times and choices were recorded. Between bumblebees, pairwise comparisons and changes of flowers contained in the towers, towers were cleaned with a 40% ethanol solution and left to dry to remove scent marks.

To test whether presence/absence of floral scent affects bumblebee selection of feeding places, we assessed the innate preference of bumblebees for towers with and without flowers (5+5=10 towers). For that purpose, different lines of flowers were used in each replicate (*n*=10). The towers without flowers were previously unused and had dampened cotton added.

In addition, we tested the innate preferences of bumblebees (*B. terrestris*, Biohelp, Austria) for some VOCs found in the *Antirrhinum* flowers: methyl benzoate, o-acetanisole, ethyl benzoate, o-acetylphenol, sabinene, decanal, and methyl cinnamate. We introduced two filter paper artificial flowers attached to 1.5ml tubes, in a 5L clean container, which was orientated upside down (Supplementary Figure S5). One artificial flower was supplemented with a VOC diluted in acetone and the other was used as control with just acetone. The same quantities of acetone for the control and the diluted VOC were used. Around 1.5ml tubes were supplied with 10μl of sucrose solution. Bees were tested individually and each bee was only tested once. Bumblebees did not repeat experiments and when bumblebees fed from sugar-supplied tubes, choices were recorded (*n*≥10).

#### Experiments With Thrips

Field populations of thrips (*F. occidentalis*) collected in Murcia (Spain) were reared for two generations in the lab (Espinosa et al., 2002). Thrips used were females and flower-naïve. For experiments with flowers, we used new 0.5L plastic boxes with one flower (without stamens) from each line (Supplementary Figure S6A). We added 30 thrips in each pairwise analysis (*n*: 4–9). In the case of stamens, they were introduced in Petri dishes (Ø=14cm; Supplementary Figure S6) and 20 thrips were included in each dish (*n*: 3–5). We controlled for the effects of ambient light/Petri dish orientation. Thrips were kept inside the plastic boxes/Petri dishes for 24h, at 25°C and in 16:8h of light/dark. Then, thrips were placed in a freezer (5min) to stun them before counting the number on each flower/stamen and its immediate surroundings: 2cm distance from the flower/stamens.

To test the effect of some VOCs on thrips, we introduced two filter paper artificial flowers into 5L sealed containers. One flower was supplemented with a VOC (diluted in acetone) and the other with acetone (Supplementary Figure S7). VOCs tested were: α-farnesene, methyl benzoate, o-acetanisole, cinnamyl alcohol, methyl cinnamate, and β-myrcene. Concentrations used are indicated in Table 2. About 10 thrips were introduced and left inside for at least 60min. Then, we counted the number of thrips in each artificial flower.

**Table 2:** Effect of isolated VOCs tested with each organism, at indicated concentrations.

Organism	VOC	Concentration (ppm)	Volume (μl)	No. VOC	No. control	Replicates	Chisquare (*χ*^2^)	Chisquare (value of *p*)
Bumblebees	Methyl benzoate	1,000	10	12	4	16	4.000	**0.046**
	o-acetanisole	1,000	10	3	11	14	4.571	**0.033**
	o-acetanisole	100	10	5	6	11	0.091	0.763
	o-acetanisole	10	10	5	6	11	0.091	0.763
	Ethyl benzoate	1,000	10	5	9	14	1.142	0.285
	o-acetylphenol	1,000	10	9	5	14	1.142	0.285
	Sabinene	1,000	10	8	6	14	0.286	0.593
	α-farnesene	1,000	10	9	5	14	1.143	0.285
	Decanal	1,000	10	8	6	14	0.286	0.593
	Methyl cinnamate	1,000	10	8	6	14	0.286	0.593
Thrips	α-farnesene	1,000	5	49	52	101	0.089	0.765
	Methyl benzoate	1,000	5	29	31	60	0.067	0.796
	o-acetanisole	1,000	5	27	26	53	0.019	0.891
	Cinnamyl alcohol	1,000	5	19	19	38	0	1.000
	Methyl cinnamate	1,000	5	15	17	32	0.125	0.724
	β-myrcene	1,000	2	21	20	41	0.0024	0.876
	β-myrcene	1,000	5	46	38	84	0.762	0.383
	β-myrcene	1,000	10	39	29	68	0.225	0.225
	β-myrcene	no dilution	10	8	17	25	0.072	0.072
	^*^β-myrcene	no dilution	100	9	2	11	4.456	**0.035**
	^**^β-myrcene			4	13	17	2	**0.029**

Number (No.) VOC and No. control show the number of individuals of each organism, which chose the VOC being tested or the control, respectively. In the experiments with thrips, individuals, which did not choose either the VOC or control were discounted. Number of replicates are indicated along with Chi-square *p*-values. In β-myrcene experiments: single asterisk (^*^) indicates results for dead thrips found and double asterisks (^**^) indicate results for thrips alive on artificial flowers. Significant results (*p*<0.05) are in bold.

#### Experiments With Humans

Plant experts (14 women and 16 men) were recruited amongst horticulturists from the Cambridge University Botanic Garden, and plant scientists from the University of Cambridge – Department of Plant Sciences. The experts were asked to interact with each studied pairing of *Antirrhinum* flowers. Three flowers of each pairing were placed at an individual station in an indoor area at the Botanic Garden. Each pairing and each station were identified by a code. There were 11 stations in total and experts were asked to complete a survey with one question addressing each station. Experts answered the questions in the survey by moving between stations haphazardly until all were completed. The survey was divided into two parts, a preference assessment of whole flowers presenting multimodal displays and a preference assessment of the isolated scent of flowers. In stations testing preferences for whole flowers, the experts were instructed to look at and smell all replicates of each pair (Supplementary Figure S8A). They then answered the question “If you had to choose flowers from one group only, which flowers would you choose to grow?”, by writing down the code of flowers. For the scent-only part, experts were instructed to smell pots, in which three flowers of each pairing were hidden. Pots were covered by a fabric mesh allowing scent to escape but impeding sight (Supplementary Figure S8B). Experts then answered the question “If you had to choose one type of flower only, which flower would you choose to grow?”. An additional scent preference test was performed comparing containers with flowers and without flowers, to test if the experts prefer places with floral scent or not. All experts completed the surveys alone, and not at the same time.

### Statistical Analyses

Statistical analyses were performed in R (R Core Team, 2018) using version 3.3.3. For experiments, in which an individual represented one choice, preferences were assessed by *χ*^2^ Goodness of Fit test (Pérez-Hedo et al., 2015): thrips (flowers and artificial flowers), humans, and bumblebees with artificial flowers. In the case of bumblebee experiments with flowers, we calculated the proportion of choices made for each line by each bee, and analysed preferences for each pairwise comparison using a Wilcoxon test (Strauch et al., 2014). We did not correct for multiple comparisons at this stage and these individual tests should be considered in this light; however, our main conclusions and subsequent consideration of the results are drawn from the combined analyses described below.

In order to be able to compare preferences of organisms for each of the lines, we standardised the preference for the lines relative to the preference for *A. majus*, since all preferences were tested in pairwise with *A. majus*. Therefore, we calculated the effect size of the responses to each line relative to *A. majus* as log response ratio: [L=ln(X̅_E_/X̅_C_)], with X̅_E_ as the mean response of the organism to *A. linkianum*, RIL 112, RIL 113, RIL 9, or RIL 80 and X̅_C_ as the mean response to *A. majus* (Supplementary Table S2; Hedges et al., 1999; Junker and Blüthgen, 2010). Effect sizes range from 1 to −1, with positive values indicating a higher preference of the organism for the line under consideration compared with *A. majus*.

Euclidean distances were assessed by using the R package *vegan* (Oksanen et al., 2019) for colour, floral sizes, and scent (*vegdist*), and GEOMORPH for the morphology (*gpagen*) based on the Procrustes coordinates (Dryden and Mardia, 1993; Lockwood et al., 2004). Non-metric multidimensional scaling (NMDS, *vegan: metaMDS*) was used to analyse multivariate traits: colour, floral sizes, scent, and morphology. Due to low dimensionality, we used classical MDS (*vegan: cmdscale*) to ordinate pollen data. Ordinations (NMDS and MDS) represent how similar the analysed flowers are for a given trait. Thus, the closer two points are, the more similar is their multidimensional phenotype. We obtained environmental vectors (*vegan: envfit*), referred to as vectors in the manuscript, and fitted them into ordinations. These vectors represent the correlations between the effect sizes of each organism and the multidimensional trait under consideration. Thus, ordinations represent how similar/dissimilar flowers are for a given trait, and if the preferences of animals were significantly correlated with that trait. We used Pearson’s correlations with trichome density and vectors representing the preferences for flowers of the different animals.

A machine-learning algorithm (*randomForest* package, RF) was used to pinpoint VOCs that best explain the preferences of studied animals for floral scents (Breiman, 2001; Helletsgruber et al., 2017). We performed an RF for regression using the effect sizes of each line as the dependent variable, and the VOC emission of flowers from each line as the explanatory variable. We used the square root of the total number of variables as *m_try_*, and grew a total of *n_tree_*=20,000 trees. In regression tasks, function *importance* provides the mean square error (%IncMSE), which informs about the variables that explain the preferences of animals. We used %IncMSE for the preferences of bumblebees and humans for the scent of flowers with stamens and the preferences of thrips for the scent of flowers without stamens and stamens separately.

Bipartite network analysis was performed with *bipartite* package (Dormann, 2011). Coefficients of determination (*r*^2^) from vectors, in each ordination, and Pearson’s correlations were used to create a bipartite network between organisms and groups of floral traits. We used the *r*^2^ of significant (*p*<0.05) results for each trait vs. each organism. Displayed width of edges in the bipartite network is proportional to coefficients of determination *r*^2^ of significant traits for each organism.

Function *rcorr* with default values (R package *Hmisc*; Harrell et al., 2019) was used to obtain a correlation matrix between all phenotypic traits (except colour and morphology). We represented significant (*p*<0.05) correlations using corrplot R package (Wei and Simko, 2021) and function. Phenotypic data used for the correlation matrix is available in Supplementary Table S3.

## Results

We tested the preferences of bumblebees, human plant experts, and thrips for different *Antirrhinum* flowers. Parental *A. majus* flowers are larger and, to human vision, lighter in colour than those of parental *A. linkianum*. Out of the four RILs studied, RIL 112 is more similar in colour and shape to *A. linkianum* than the other RILs. RILs 113 and 9 look similar, whereas RIL 80 is notably different to all other lines studied, since it is smaller and completely white (Figure 1).

### Preference Assessments

**Figure 3:**
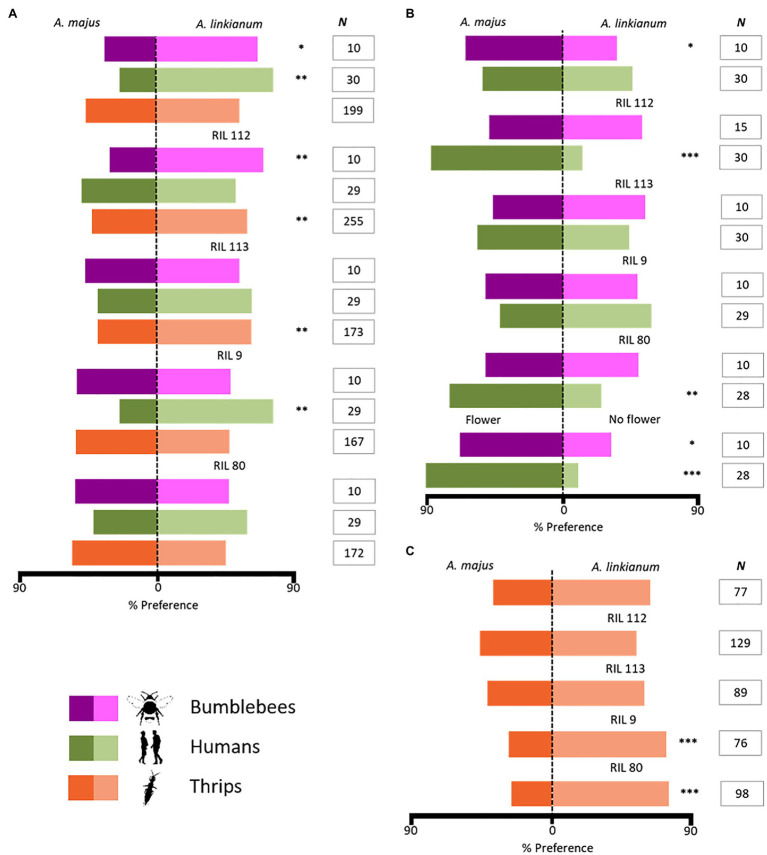
Preference results of animals for flowers of *Antirrhinum*. **(A)** Preferences of bumblebees, humans, and thrips for whole flowers. **(B)** Preferences of bumblebees and humans for just the scent of flowers. **(C)** Preferences of thrips for stamens of flowers. All lines were compared with *A. majus* (except for the isolated scent from flower vs. no flower comparison in **B**). Bumblebees (magenta), humans (green), and thrips (orange). Asterisks indicate the level of significance in statistical results: (^*^) *p*≤0.05, (^**^) *p*≤0.01, and (^***^) *p*≤0.001. Non-significant results are not indicated. Number of replicates (*N*) per experiment is indicated.

The assessment of the preferences of bumblebees for whole flowers showed that they made more choices for *A. linkianum* (Wilcoxon *v*=21, *p*=0.035) and RIL 112 flowers (Wilcoxon *v*=45, *p*=0.009), than *A. majus* blossoms. *Antirrhinum linkianum* (*χ*^2^=8.533, *p*=0.003) flowers were also significantly more appealing to humans, along with RIL 9 (*χ*^2^=7.759, *p*=0.005). In contrast, thrips showed preferences for visiting more flowers of RILs 112 (*χ*^2^=7.251, *p*=0.007) and 113 (*χ*^2^=9.717, *p*=0.002; Figure 3A).

Contrastingly, experiments testing the preferences for floral scent in isolation indicate that bumblebees made more choices for feeders (towers) with the scent of *A. majus* vs. *A. linkianum* (Wilcoxon *v*=28, *p*=0.021). Humans preferred to grow flowers with the scent of *A. majus* compared to flowers of RILs 112 (*χ*^2^=16.133, *p*<0.001) and 80 (*χ*^2^=7, *p*=0.008). Furthermore, both bumblebees and humans showed preferences for floral scented rather than non-scented towers (Wilcoxon *v*=42, *p*=0.021 and *χ*^2^=19.2, *p*<0.001, respectively; Figure 3B).

Finally, thrips preferred to visit stamens from RILs 9 (*χ*^2^=8.895, *p*<0.001) and 80 (*χ*^2^=14.735, *p*<0.001; Figure 3C). However, when they interacted with flowers from RILs 9 and 80, which did not contain stamens, they did not show preferences for them (RIL 9: *χ*^2^=0.485, *p*=0.486; RIL 80: *χ*^2^=1.884, *p*=0.170; Figure 3A).

Results for bumblebees suggest that visual cues override scent emission when guiding their preferences for floral visitation (Figures 3A,B). Results for thrips indicate that their attraction differs between the different floral parts, since they showed preferences for either the stamens of flowers, or the flowers independently of the stamens (Figure 3). Altogether, bumblebees showed preferences for parental species and RIL 112, whereas humans generally preferred parental species and RIL 9. In contrast, thrips preferred to visit all RILs over the parental lines.

### Deconstructing the Floral Phenotype in Relation to Attraction

#### Colour, Morphology, and Floral Sizes

To the human eye, lines used in this study range from dark pink to white, with parental *A. majus*, RIL 113 and, to a lesser extent, RIL 9, presenting veined patterning in the floral lobes. In addition, *A. majus*, *A. linkianum*, and RIL 113 all present yellow palates (Figure 1). NMDS ordination of colour data clearly separates *A. linkianum* and RIL 80 from each other as well as from the other lines. In contrast *A. majus*, RIL 9, RIL 112, and RIL 113, are not clearly separated by colour in the ordination (Supplementary Figure S9A). Vectors fitted in ordinations indicate that this floral attribute is statistically significant for bumblebees (*r*^2^=0.356, *p*=0.001), humans (*r*^2^=0.269, *p*=0.001), and thrips (*r*^2^=0.168, *p*=0.001). Bumblebees and humans preferred the dark-pink lines *A. linkianum* and RIL 112 as the vectors pointed towards these lines in the ordination. In contrast, thrips seem to prefer lines with lighter colours such as *A. majus*, RIL 113, and RIL 9 (Supplementary Figure S9A).

Ordination of Euclidean distances of morphological data distinctively separates each line (Supplementary Figure S9B). Vectors representing significant preferences associated with morphology indicate that this trait affects bumblebee foraging decisions, with the vector pointing towards flowers shaped like *A. linkianum* and RIL 112 (*r*^2^=0.354, *p*=0.001). In addition, floral morphology is also behaviourally significant for humans when they are asked to choose which type of flowers they would prefer to grow (*r*^2^=0.346, *p*=0.003), whilst it is not for thrips when they choose which flowers to visit (*r*^2^=0.010, *p*=0.872; Supplementary Figure S9B).

Finally, the ordination of floral size data clearly differentiates lines *A. majus*, *A. linkianum*, and RIL 80. In addition, vectors representing significant associations of animal preferences for flowers indicate that the size of different floral parts affect the choices of flowers of both bumblebees (*r*^2^=0.087, *p*=0.012) and humans (*r*^2^=0.230, *p*=0.001; Supplementary Figure S9C).

Whilst colour is a relevant trait for bumblebees, humans, and thrips, the morphology of flowers and the size of different floral parts affect the choices of bumblebees and humans. This indicates that very small animals such as thrips would not be so influenced by the size and shape of flowers.

#### Scent

A total of 29 different compounds were found in the scent profiles of studied flowers (Table 1). The most scented line (average total emission) was RIL 9, whilst the lowest emitter was RIL 112 (Levene test: *F*=1.608, *p*=0.173; ANOVA: *F*=6.633, *p*<0.001). Some VOCs, like (E)- β-ocimene and β-myrcene, were emitted by all lines. However, some VOCs that were not emitted constitutively in parental lines, were found in RILs, such as methyl salicylate (RILs 113, 80, and 9), benzenepropanol (RILs 112 and 9), 3,5-dimethoxytoluene (RIL 80), or linalool (RIL 9).

We used the scent composition of flowers with stamens (whole flowers) to analyse the preferences of bumblebees and humans. NMDS ordination of the scent profiles of whole flowers clearly separates the scent emission of *A. linkianum* from RIL 113 and 80, which are located at the bottom and the top of the ordination, respectively (Supplementary Figures S10A,B). In the centre of the ordination is the parental species *A. majus*, which is more similar in its scent bouquet to RILs 112 and 9 (Supplementary Figures S10A,B). We tested the preferences of bumblebees and humans for both whole flower multimodal displays (animals interacting with flowers) and just the scent of whole flowers. Thus, we fitted vectors representing preference results into two different ordinations (Supplementary Figures S10A,B).

Vectors representing the preferences of bumblebees and humans for the scent of whole flowers (Supplementary Figure S10A) indicate that this trait is important for both types of animals when they can see and smell the flowers: bumblebees (*r*^2^=0.481, *p*=0.001), humans (*r*^2^=0.375, *p*=0.001). In this case, bumblebees and humans have similar preferences for the scent of whole flowers (Supplementary Figure S10A), with vectors pointing towards *A. linkianum*.

When bumblebees and humans are unable to see the flowers, vectors representing their preferences for the scent of flowers (Supplementary Figure S10B) indicate that this trait is still relevant for them (*r*^2^=0.585, *p*=0.001 and *r*^2^=0.191, *p*=0.002, respectively). Interestingly, results for bumblebees with regard to the isolated scent of flowers show a completely diametrical configuration in the NMDS ordination, compared with the scent of flowers when they can interact with the whole flowers (Supplementary Figures S10A,B). Results indicate that visual cues prevail over scent preferences of bumblebees. In the case of humans, the direction of the vector does not change in the NMDS ordination for scent in isolation compared to the scent of whole flowers (Supplementary Figures S10A,B), revealing that scent is a trait strongly affecting the preferences of plant experts for flowers.

We analysed the scent emission of flowers without stamens. NMDS analysis of the scent profile of flowers without stamens shows the same configuration as previously described for the different lines, with *A. majus* in the centre of the ordination and *A. linkianum* at the bottom (Supplementary Figures S10A–C). Hence, the scent bouquet of flowers is barely affected by the removal of stamens (Supplementary Figures S10A,C). Fitting the vectors representing the preferences of thrips into the ordination (Supplementary Figure S10C; *r*^2^=0.267, *p*=0.001), indicates that thrips have opposing preferences for flowers than bumblebees and humans when they can see and smell whole flowers (Supplementary Figure S10A). We also analysed the scent emission of stamens removed from flowers and tested the preferences of thrips for detached stamens. Ordination of the scent emission from stamens of flowers studied generally separates each line clearly, with *A. linkianum* and RIL 112 overlapping in some points. The vector representing the preferences of thrips for stamens detached from flowers indicates that this trait is statistically significant (*r*^2^=0.220, *p*=0.016; Supplementary Figure S10D).

**Figure 4:**
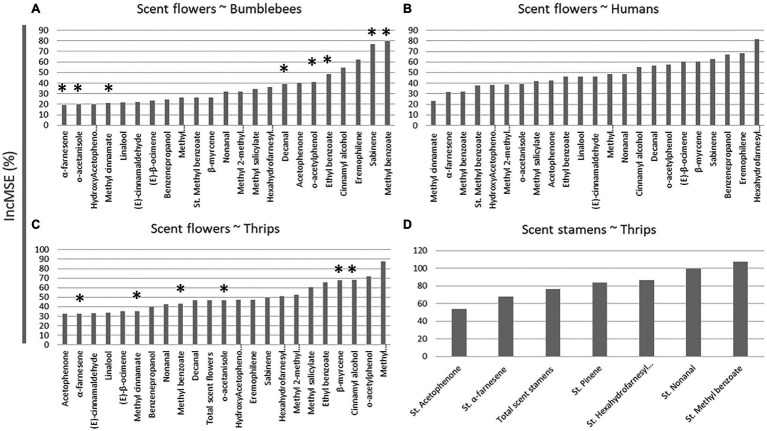
Percentage of the mean square error (%IncMSE) resulting from random forest regression analysis of the preferences of studied animals as the dependant variable and the emission of volatile organic compound (VOCs) as the explanatory variable. **(A)** Bumblebee preferences against the scent of flowers with stamens. **(B)** Human preferences against the scent of flowers with stamens. **(C)** Preferences of thrips for the scent of flowers without stamens. **(D)** Preferences of thrips for the scent of stamens. Asterisks indicate isolated volatiles that have been used for behavioural assays with bumblebees and thrips.

We used RF for the selection of the most important VOCs correlating with the effect sizes representing the preferences of studied animals. We used this approach with the results of experiments based on preferences for the isolated floral scents of bumblebees and humans (Figures 4A,B). We also used this approach for testing the preferences of thrips for the scent of flowers without stamens and detached stamens (Figures 4C,D). Results indicate that the behaviour of bumblebees might be affected by some VOCs including methyl benzoate, sabinene, eremophilene, or cinnamyl alcohol (Figure 4A). In contrast, hexahydrofarnesyl acetone, eremophilene, benzenepropanol, sabinene, β-myrcene, or β-ocimene may affect the preferences for the scent of flowers of human plant-specialists (Figure 4B). Finally, methyl hydrocinnamate, o-aceylphenol, cinnamyl alcohol, or β-myrcene may affect the behaviour of thrips towards flowers (Figure 4C), whilst methyl benzoate or nonanal may influence the decisions of thrips when choosing just stamens (Figure 4D).

We were not able to test the effect of all VOCs identified by RF on bumblebees and thrips but we were able to test the effect of some VOCs with high %IncMSE (Figure 4), for which, there are commercially available authentic standards, on the behaviour of bumblebees and thrips (Table 2). Results indicate that, in the case of bumblebees, methyl benzoate seems to be an attractant. However, o-acetanisole in high concentrations is a repellent. Testing concentrations within those between the levels of methyl benzoate (33%) and o-acetanisole (0.4%) found in the flowers (Table 1) indicates that below 100ppm o-acetanisole does not have an effect on the behaviour of bumblebees. Additionally, ethyl benzoate, o-acetylphenol, sabinene, α-farnesene, decanal, and methyl cinnamate did not show any effect under our experimental conditions (Table 2). Similarly, we tested the effect of some VOCs on the behaviour of thrips. Results obtained show that α-farnesene, methyl benzoate, o-acetanisole, cinnamyl alcohol, methyl cinnamate, and β-myrcene do not have any effect on thrips behaviour at concentrations of 1,000ppm. However, the use of pure β-myrcene may kill these insects (Table 2).

Scent is a relevant floral trait for all organisms studied. In the case of bumblebees, visual cues are more relevant than the isolated scent of flowers, methyl benzoate can be an attractant and o-acetylphenol a repellent. Human preferences for the scent of flowers do not change when they can see the flowers. On the other hand, thrips are attracted differentially by the scent emitted by the flowers and by the stamens, and their attraction towards flowers by floral scent contrasts with that of bumblebees and humans. Finally, β-myrcene can kill thrips at high doses.

#### Pollen and Trichomes

**Figure 5:**
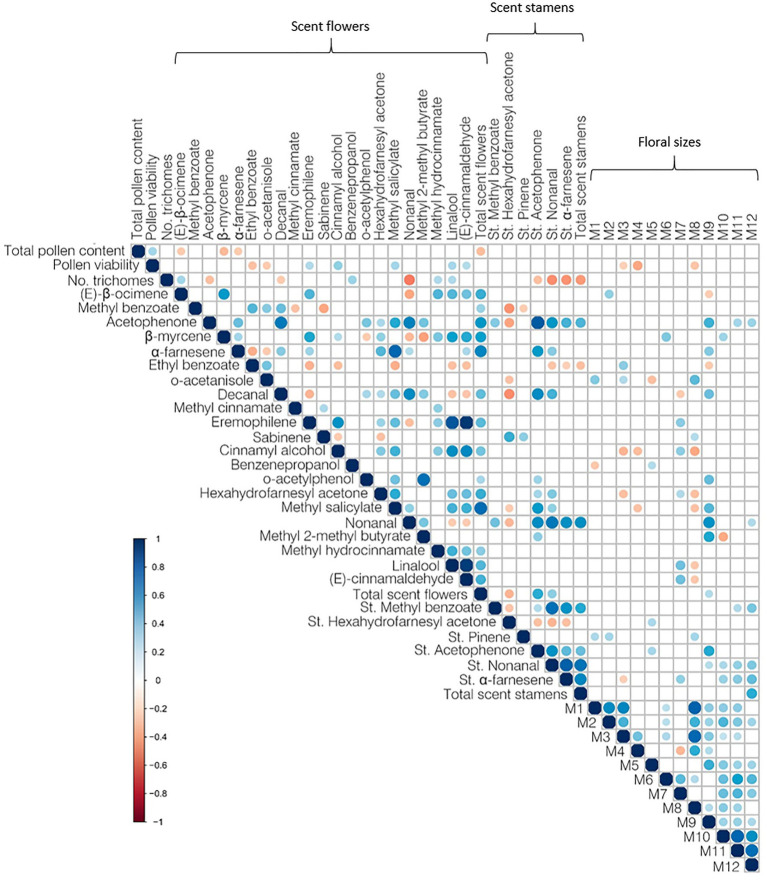
Correlogram of the correlation matrix of floral phenotypic traits: pollen features, floral scent without stamens, scent of stamens, and floral sizes. Only significant Pearson’s correlations (*p*<0.05) are plotted.

Our experiments with thrips and stamens allowed the insects to interact with the pollen and thus, potentially, develop preferences for it, which is supported by the significant results (*r*^2^=0.156, *p*=0.003; Supplementary Figure S10). Surprisingly, our results appear to indicate that human plant experts’ preferences for *Antirrhinum* flowers also were influenced by pollen traits (*r*^2^=0.131, *p*=0.022, Supplementary Figure S10) and trichome density (*r*=0.299, *r*^2^=0.089, *p*=0.019; Supplementary Figure S9B). Since humans did not touch flowers to open them and see the trichomes or the pollen closely, this result might be caused by the correlation of these traits with other floral attributes, such as the scent of flowers (Figure 5).

Although, our results appear to indicate that pollen is a relevant trait for thrips and humans, whereas trichomes are important for plant experts, careful interpretation of these results in the context of the experimental design suggests that they represent coincidental correlations.

#### Relative Contribution of Phenotypes to the Choices of Animals

**Figure 6:**
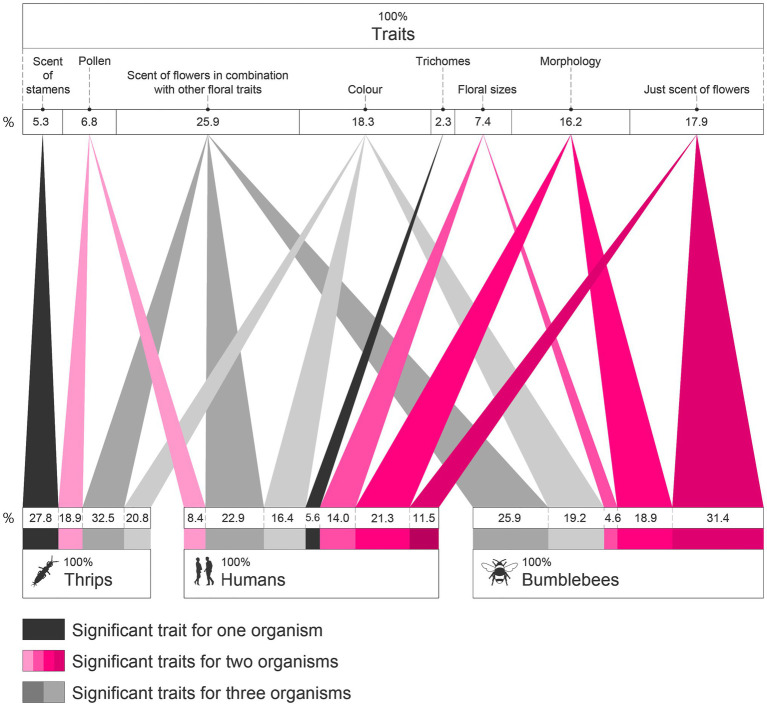
Bipartite network of statistically significant relations of the preferences of bumblebees, humans, and thrips for groups of floral traits. Colour of edges represents: traits relevant for all organisms (grey), for two organisms (pink), or for just one organism (black). Only significant traits (*p*<0.05) are represented for each organism. Width of edges is proportional to *r*^2^ for each organism and trait. Percentages indicated are calculated based on the width of each trait. In the upper part of the graph, percentages represent the relative importance of each trait analysed for the three kind of organisms together. In the lower part of the graph, percentages represent the relative importance of traits for each kind of organism separately (thrips, humans, and bumblebees).

Bipartite network analysis of significant results (*envfit*, *cmdscale*, and correlations of univariate traits) reflects the relative importance of analysed floral traits for the three groups of organisms studied (Figure 6). Preferences seem to be multimodal responses with increasing complexity regarding the number of traits involved, from thrips with the lowest number of traits, to humans with the most. From the total trait spectrum studied, the most important traits affecting all organisms seem to be floral scent and colour. Choices of humans and bumblebees show responses towards the size of floral parts, the morphology of flowers, and the isolated scent of flowers, whilst the choices of humans and thrips seem to be affected by (or correlated with) pollen features. Our data suggest that the responses of thrips towards flowers are more affected by their scent and the scent of stamens. Whereas the most important traits for humans seem to be the scent of flowers and their morphology, bumblebees seem to be more influenced by the scent of flowers, either in combination with other traits or in isolation. Correlations found amongst floral phenotypic traits might underlay some of these associations (Figure 5), such as correlations between pollen viability and several scent compounds. Altogether, bumblebees and humans seem to be attracted by more similar floral attributes than the floral traits that are relevant for thrips with regard to *Antirrhinum* flowers.

## Discussion

The ways in which humans interact and interfere with nature and plants are shaped by discernment through multiple sensory modalities (Storms and Zyda, 2000; Lindemann-Matthies et al., 2010). As our results indicate, human selection of ornamental flowers is also the consequence of decisions based on several different floral attributes. Similarly, mutualistic and antagonistic flower visitors might favour or disfavour multimodal floral traits (Katzenberger et al., 2013; Terry et al., 2014), which affects floral selection and evolution (Ramos and Schiestl, 2019). However, the traits affecting the behaviour of animals may vary between different organisms such as humans, bumblebees, and thrips. Here, we aimed to gain a more complete understanding of the mechanisms by which specific floral traits could impact plant fitness. We found that in general, humans and bumblebees share preferences towards floral traits, and that these preferences contrast with those of thrips.

### Floral Traits Affecting the Choices of Humans, Pollinators, and Pests

Findings reported here are similar to previous studies informing about the relevance of colour (Odell et al., 1999; Sampson and Kirk, 2013; Moyroud et al., 2017), floral sizes, morphology (Mainali and Lim, 2011; Moyroud and Glover, 2017), pollen (Kirk, 2009; Wilmsen et al., 2017), and scent (Suchet et al., 2010; Abdullah et al., 2014; Larue et al., 2016) for the attraction of bumblebees and thrips. We additionally examined the preferences of humans for the same floral phenotypes, testing whether human preferences match the preferences of the insects. Our study indicates that floral colour, morphology, and scent might be relevant traits guiding the selection of flowers by humans. These attributes have previously been observed as being important for humans and, for example, used in marketing (Bruce et al., 2003; Morrin, 2011).

We were able to directly assess the effect of some single floral traits, such as scent, isolated VOCs or detached stamens from flowers. However, the relative importance of distinct floral traits should be tested with comparative choice experiments to gauge the relative effects of all traits. Something that impedes discriminating relative effects in a multimodal scenario is the correlation between variables. For instance, our results indicate that the size of floral parts, pollen features, or the density of trichomes might influence the selection of flowers by humans. The size of floral parts can be related to their morphology (Moyroud and Glover, 2017), and that might partially explain why these traits seem to be important for both bumblebees and humans. Similarly, correlation of trichome abundance and pollen features with other floral traits, measured and not measured, could underlie the counterintuitive human preferences for these traits.

Herbivores are known to change floral traits, which affects pollinator behaviour, and thus, the community dynamics (Rusman et al., 2019). Consequently, studies working with flowers attached to plants, as well as the synchronised visitation of thrips and bumblebees, may yield differing results.

### Traits Under Pressure for Selection

Over the past 200 million years, the evolution of flowering plants (Li et al., 2019) has been guided by their interactions with the floral visitors commonly investigated by the scientific community, such as pollinators, herbivores, microbes, or natural enemies of herbivores (Armbruster, 1997; Herrera et al., 2002; Junker and Tholl, 2013; Borghi et al., 2017; Knauer and Schiestl, 2017). Much more recent is the selection of plants exerted by humans, present for just a few millenia (8.000–10.000years; Milla et al., 2015). In the case of ornamental plants and humans, this interaction has been proposed to be mutualistic (Wilson et al., 2016). At least in the context of these experiments, humans exert positive selection since they choose the flowers that they would prefer to grow. Our study suggests that humans and bumblebees have more similar preferences towards floral traits compared to those of thrips. This finding suggests that human *Antirrhinum* floral selection, for aesthetic reasons, could enhance the selection of phenotypes more attractive to bumblebees and less appealing to thrips. Correspondingly, selective forces exerted by bumblebees may boost the selection of flowers by plant breeders, whilst reducing visitation of thrips. When multiple selective forces are present, evolution of floral traits is not straightforward. Indeed, plant size, several volatiles such as methyl benzoate, p-anisaldehyde, and benzyl nitrile have been found to evolve rapidly in response to two pollinators, bumblebee, and hover flies in *Brassica* (Gervasi and Schiestl, 2017). However, the actual composition of volatiles differs significantly indicating a specificity of scent profile changes in response to differing pollinators. Furthermore, using the same system it has been shown that floral attractiveness i.e., they were more fragrant and displayed larger flowers when evolving in the presence of bumblebees as pollinators. In contrast, when plants grow in the presence of bumblebees and the hervibore *Pieris brassicae* (Ramos and Schiestl, 2019, 2020), volatile evolution, production of glucosinolates and autogamy evolve differently. The current emerging hypothesis is a possible coevolution of floral and defense traits. In our case, the differing choices of bumblebees and thrips indeed indicate a basic level of complexity whereupon evolutionary forces may act.

Finally, plants displaying floral phenotypes appealing to thrips might be at a competitive disadvantage due to both being less attractive to beneficial selectors and relatively more visited by herbivores. Remarkably, parental species *A. majus* and *A. linkianum* possess traits relevant for the fitness of the species, being more attractive to beneficial selectors than to antagonists.

### Implications of the Study: Agricultural Perspectives

Domesticated plants are the result of the directed artificial selection of plants by humans and the natural selection under cultivation exerted by beneficial visitors and antagonists (Milla et al., 2015). The study of plant traits that can enhance the survival and quality of crops, by attracting pollinators and repelling pests, is very important from an agricultural perspective. For instance, some studies have reported the avoidance of tomato flowers in greenhouses by bumblebees due to a deterrent effect of flowers (Whittington et al., 2004; Morse et al., 2012). Our results regarding the attractive effect of methyl benzoate and the repellent effect of o-acetanisole could interest breeders seeking to improve pollination of greenhouse crops (Morse et al., 2012; Ruiz-Hernández et al., 2017). On the other hand, drawbacks derived from pesticide use (Gierer et al., 2019; Varah et al., 2020) could potentially be replaced by growing naturally pest-repellent plants or the use of auxiliary plants to control thrips populations in greenhouses. Moreover, the use of high doses of β-myrcene could be a resource to control thrips on crops (Terry et al., 2014). Finally, in the case of ornamental plants, human preferences are the main driving factor selecting flowers. However, a number of factors affect the human perception of flowers such as gender, cultural background, or education (Kendal et al., 2012). Therefore, for studying human preferences under a market niche perspective (or evolutionary context) such factors should be considered.

## Conclusion

Our work suggests that instead of a single trait playing a key role in the selection of flowers, there are several floral traits interacting with different floral visitors. The relative importance of floral traits for floral visitors may change with different plant and/or visitor taxa. However, our work shows that the interactions of insects and humans with floral phenotypes could ultimately drive the evolution of flowering plants in natural and human-influenced environments. Our comparisons of the floral preferences of humans and insect flower visitors represent a novel approach that yields intriguing insights into how plant breeders may inadvertently influence insect-flower interactions.

## Data Availability Statement

The original contributions presented in the study are included in the article/Supplementary Material; further inquiries can be directed to the corresponding author.

## Ethics Statement

The studies involving human participants were reviewed and approved by Ethical Committee from the Universidad Politécnica de Cartagena (CEI19_013). Written informed consent for participation was not required for this study in accordance with the national legislation and the institutional requirements.

## Author Contributions

VR-H, PG, JW, PB, BG, RJ, and ME-C planned and designed the research. VR-H, LJ, AR-G, SA, JP, AL-Z, JB, SE, SB, and CT-R performed the experiments with plants and animals. VR-H, LJ, SA, JP, LM-R, and RJ analysed the data. VR-H and RJ wrote the manuscript. All authors contributed to the article and approved the submitted version.

## Conflict of Interest

The authors declare that the research was conducted in the absence of any commercial or financial relationships that could be construed as a potential conflict of interest.

## Publisher’s Note

All claims expressed in this article are solely those of the authors and do not necessarily represent those of their affiliated organizations, or those of the publisher, the editors and the reviewers. Any product that may be evaluated in this article, or claim that may be made by its manufacturer, is not guaranteed or endorsed by the publisher.
