# Supplementary material for: Humans Share More Preferences for Floral Phenotypes With Pollinators Than With Pests
Source: Front Plant Sci. 2021 Aug 23;12:647347. doi: 10.3389/fpls.2021.647347 (PMC8419516; doi:10.3389/fpls.2021.647347)
Supplement: Supplementary file 1 [file Data_Sheet_1.zip › Supplementary Materials.docx]

Supplementary Material

# Supplementary Figures


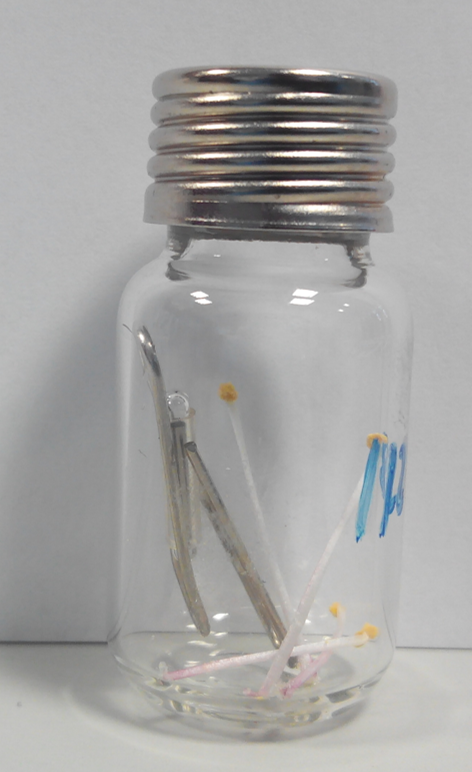


**Supplementary Figure 1.** Set-up for the collection of VOCs emitted by stamens. 10ml headspace vials were used. Stir bars were attached to paper clips to avoid their direct contact with stamens. Non- biological materials were cleaned with distilled water and ethanol 96% and dried before VOCs analysis.


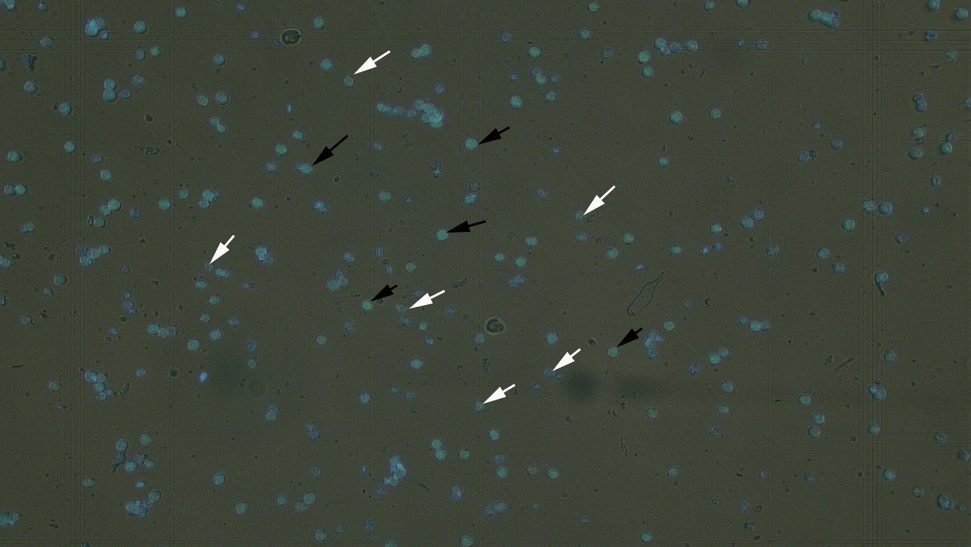


**Supplementary Figure 2.** Pollen viability determination with the fluorescein diacetate staining method. Black arrows indicate viable pollen and white arrows non-viable pollen.


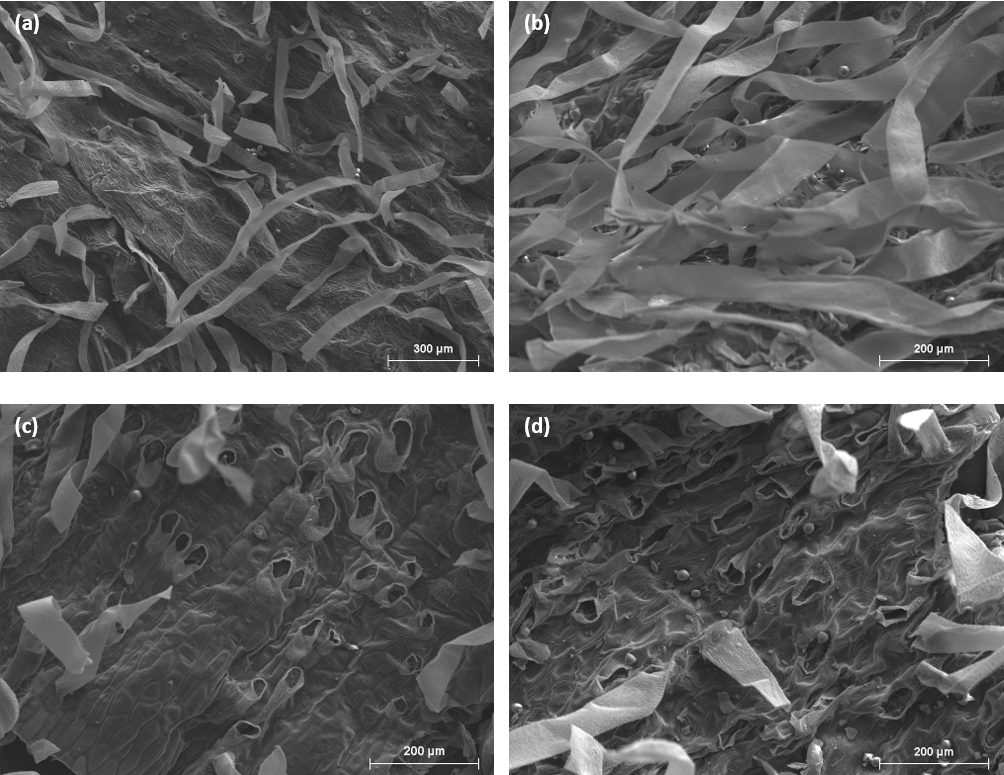


**Supplementary Figure 3.** Density of trichomes analysis. Due to the abundance and length of trichomes (a-b), we had to mechanically remove trichomes (c-d) and use the bases of trichomes as a proxy for trichomes density.


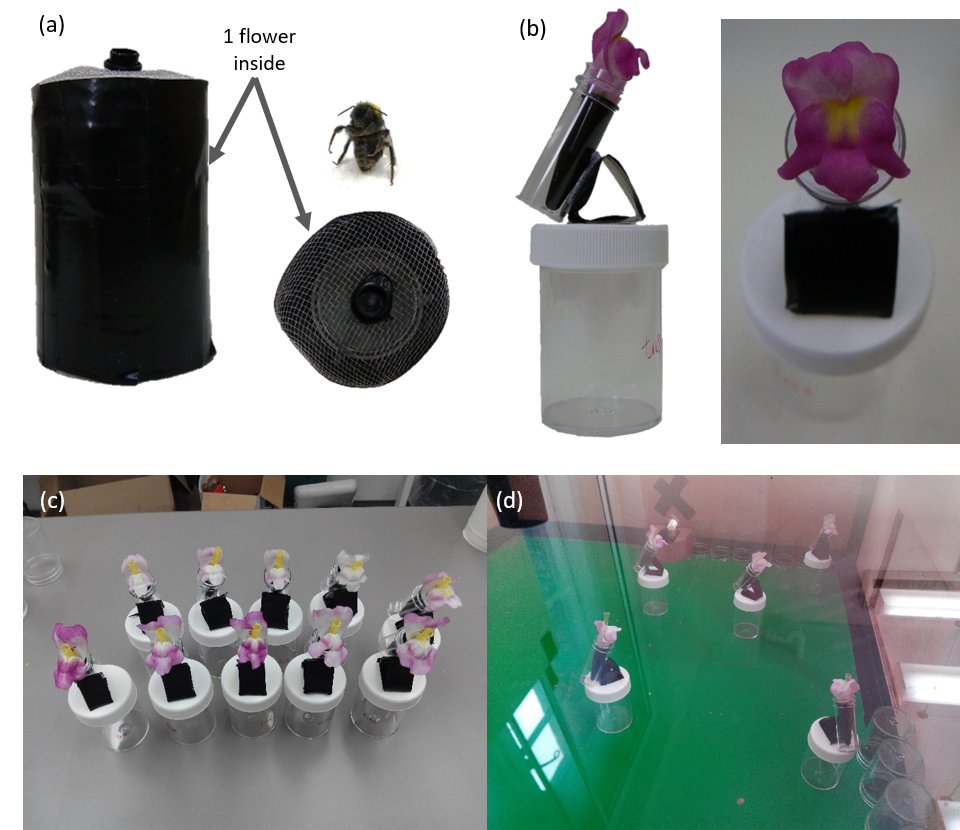


**Supplementary Figure 4.** Experimental set-up used to test the preferences of bumblebees for flowers of *Antirrhinum*. (a) Floral scent in isolation, detailed set-up. (b) Whole flowers, detailed set-up. Flowers were postioned approximately at an inclination of 60°, using a black platform, to simulate the position of flowers in the plant. (c) Flowers with yellow pipette tips supplied with 20µl of sugar solution. (d) Pseudo-random display of flowers in the flight arena.


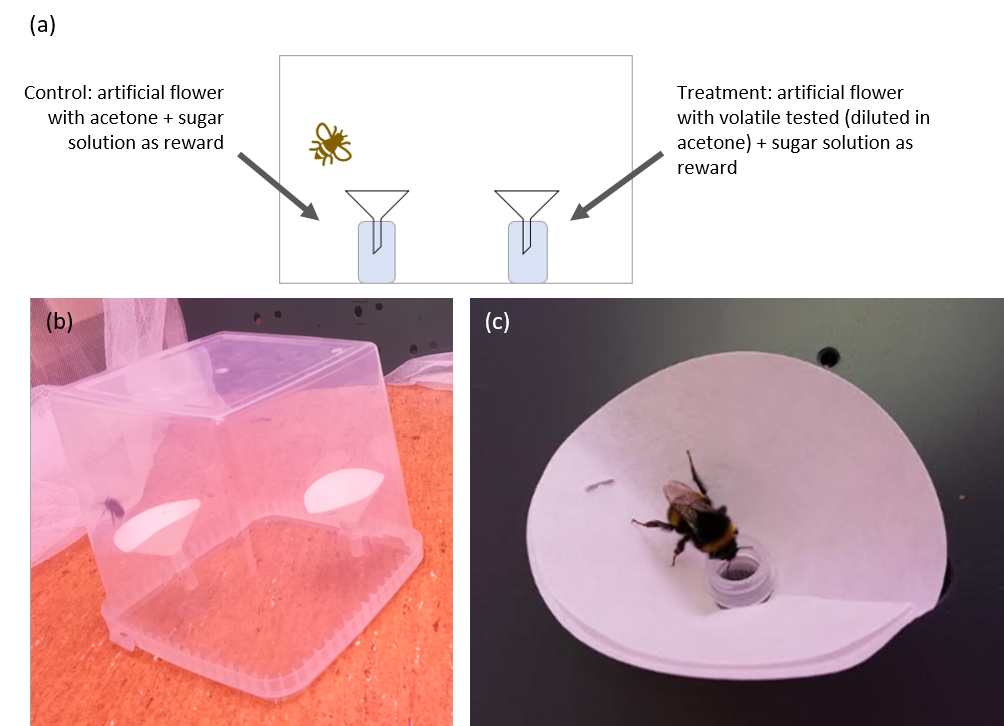


**Supplementary Figure 5.** Experimental set-up used for testing the preferences of bumblebees for isolated VOCs. The first feeding-flower choice of every bumblebee was recorded. (a-b) Diagram and photo of the set-up, respectively. (c) Photo of bumblebee feeding on an artificial flower.


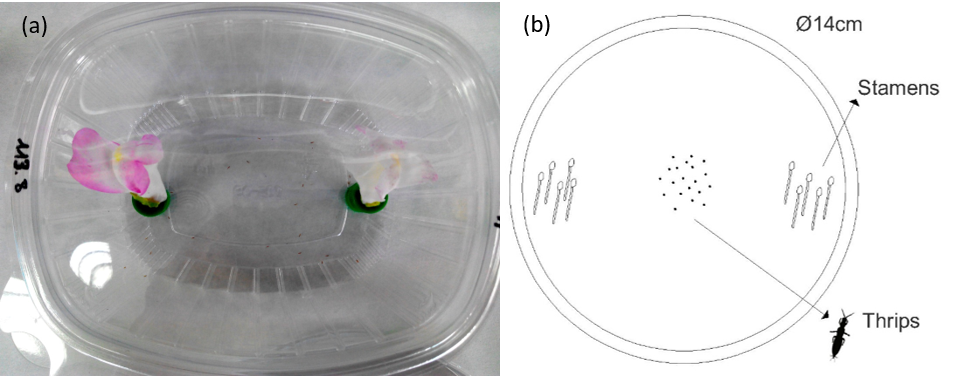


**Supplementary Figure 6.** Experimental set-up used for the assessment of the preferences of thrips for (a) flowers without stamens and (b) the stamens of *Antirrhinum* sp. Thrips were placed in the center of the container and allowed to move freely within it for 24h.


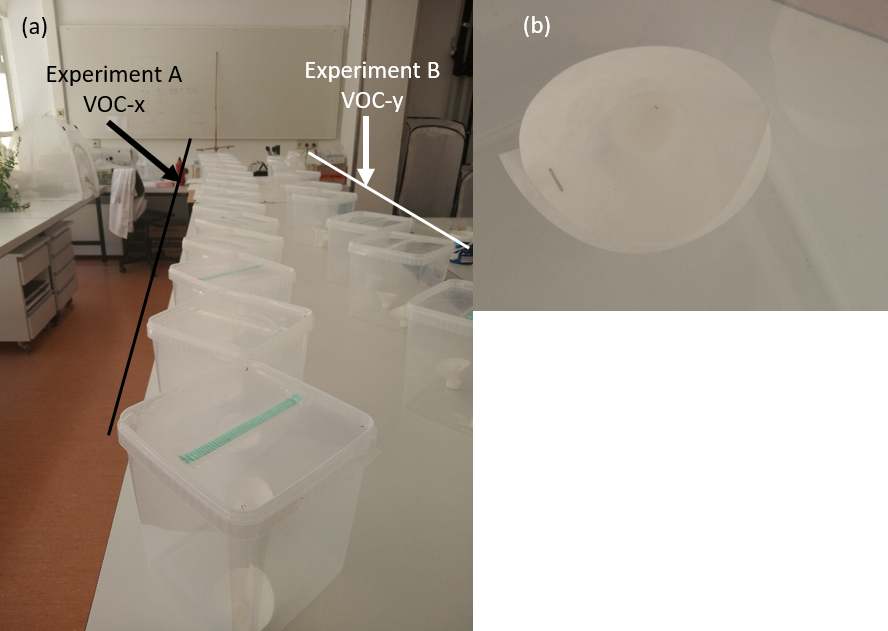


**Supplementary Figure 7.** Experimental set-up used for the assessment of the preferences of thrips for isolated VOCs. (a) General view of the set-up and control for the light/shadow ratio received by each container and experiment. (b) Detail of filter-paper artificial flower.


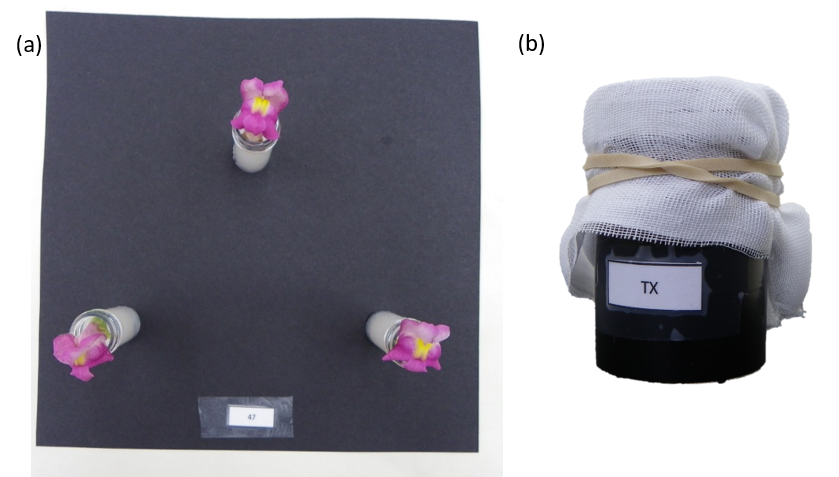


**Supplementary Figure 8.** Experimental set-up used to test the preferences of humans for whole flowers of *Antirrhinum* (a) and the floral scent in isolation of the same flowers (b).


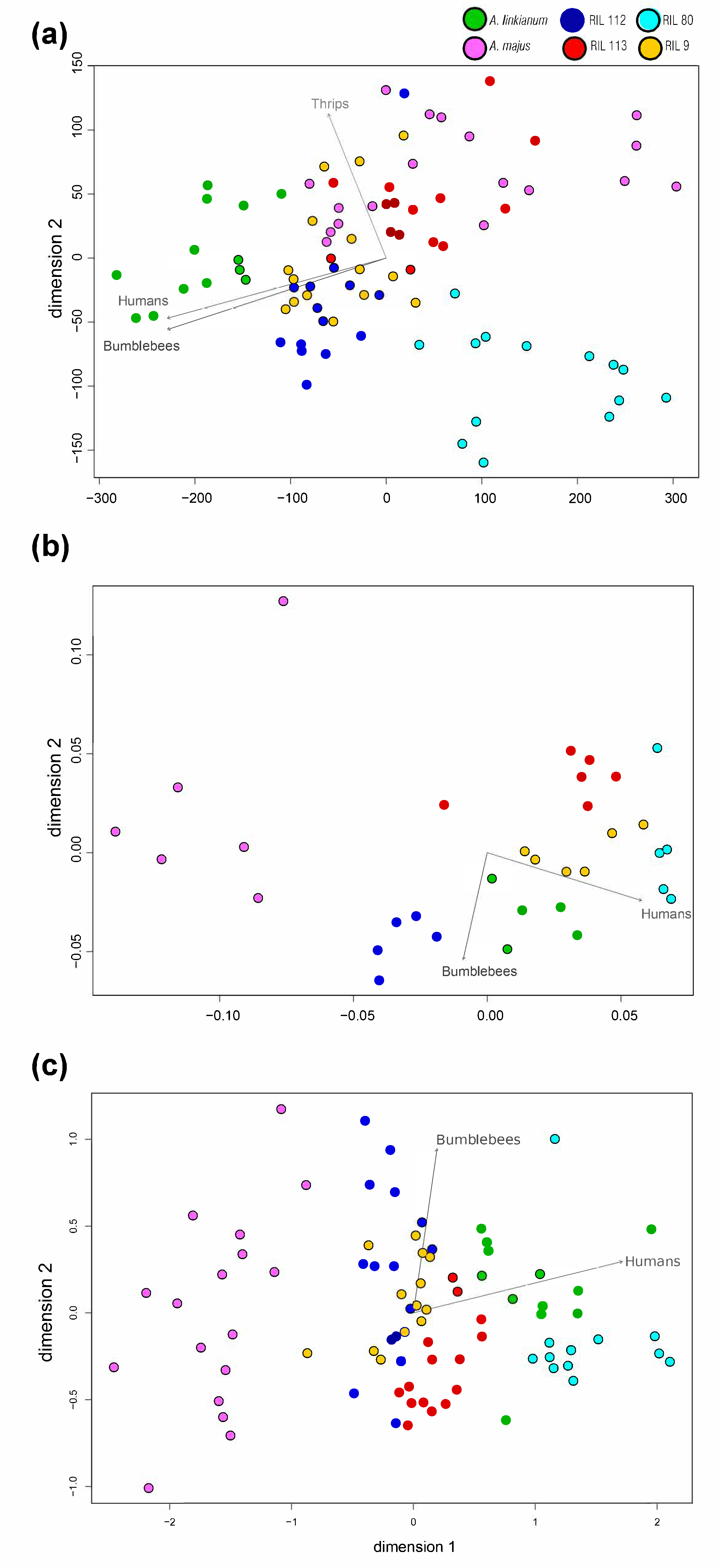


**Supplementary Figure 9.** Ordination of colour **(A)**, morphology **(B)** and the floral size **(C)** of different parts from Antirrhinum flowers (see Figure 2). Ordinations represent the two dimensions of non-metric multidimensional scaling (NMDS) representation of Euclidean distances between replicates and lines. Vectors represent significant preferences of animals for the traits represented by the ordination (see Figure 3).


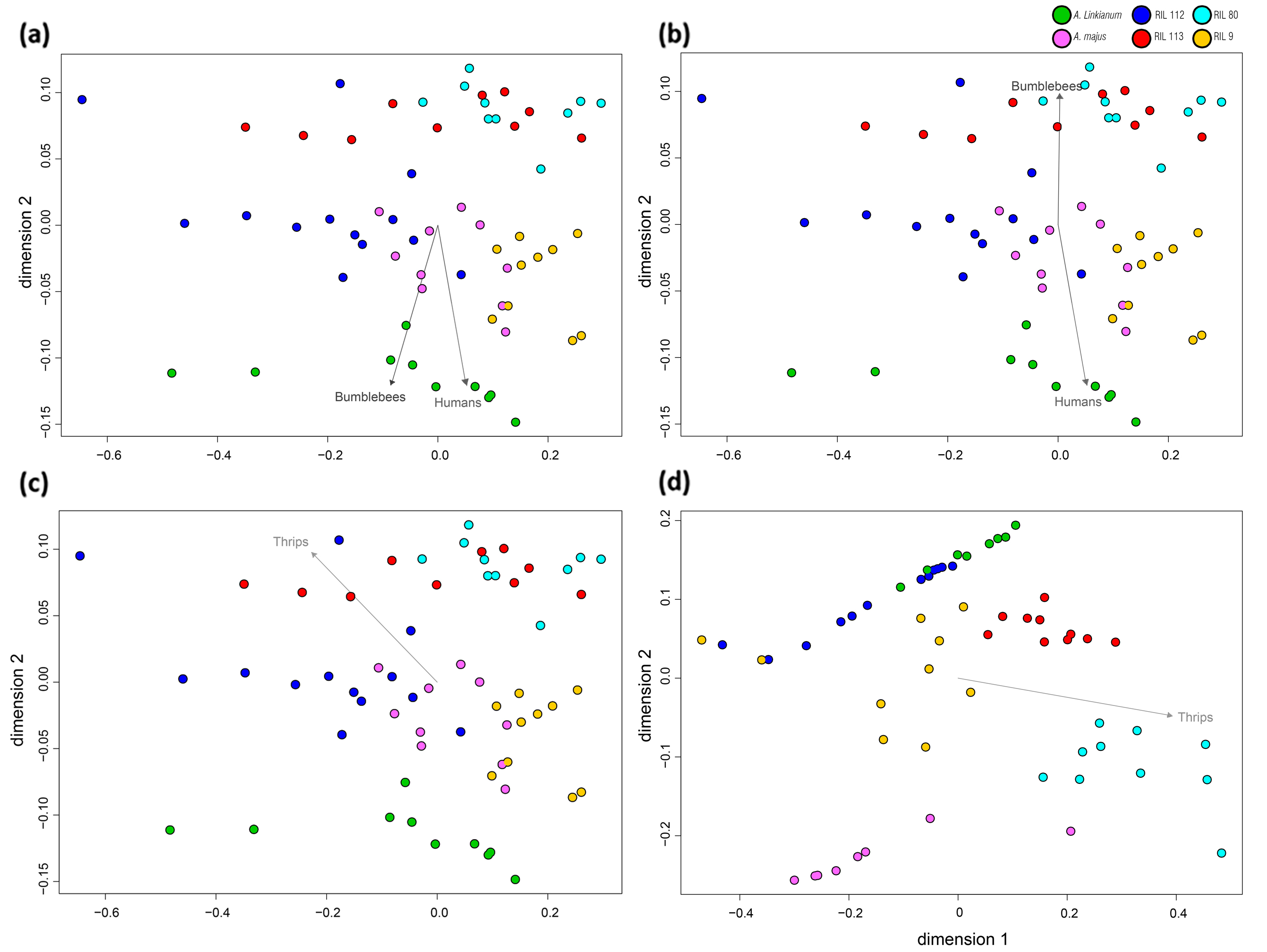


**Supplementary Figure 10.** Ordination of the scent emission of whole flowers **(A-B)**, flowers without stamens **(C)** and stamens **(D)** from *Antirrhinum*. Ordinations represent the two dimensions of non-metric multidimensional scaling (NMDS) representation of Euclidean distances between replicates and lines. Vectors represent significant preferences of animals for scent emissions (see Figure 3). Vectors plotted in **(A)** represent the preferences of bumblebees and humans for the scent of flowers with stamens, in combination with other floral traits. Vectors plotted in **(B)** represent the preferences of bumblebees and humans for floral scent in isolation from other traits (flowers hidden from view). Vectors plotted in **(C)** represent the preferences of thrips for the scent of flowers without stamens, in combination with other floral traits. Vectors plotted in **(D)** represent the preferences of thrips for the scent of stamens, in combination with other stamen traits.


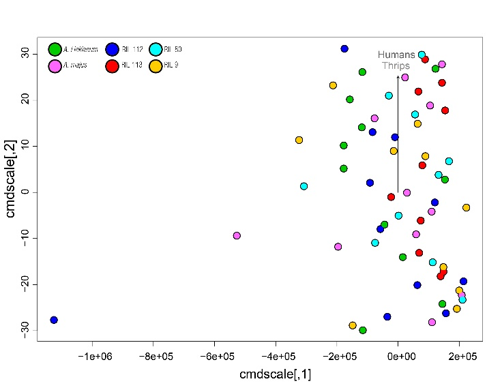


**Supplementary Figure 11.** Cmdscale ordination of pollen traits and environmental fitting of significant preferences.


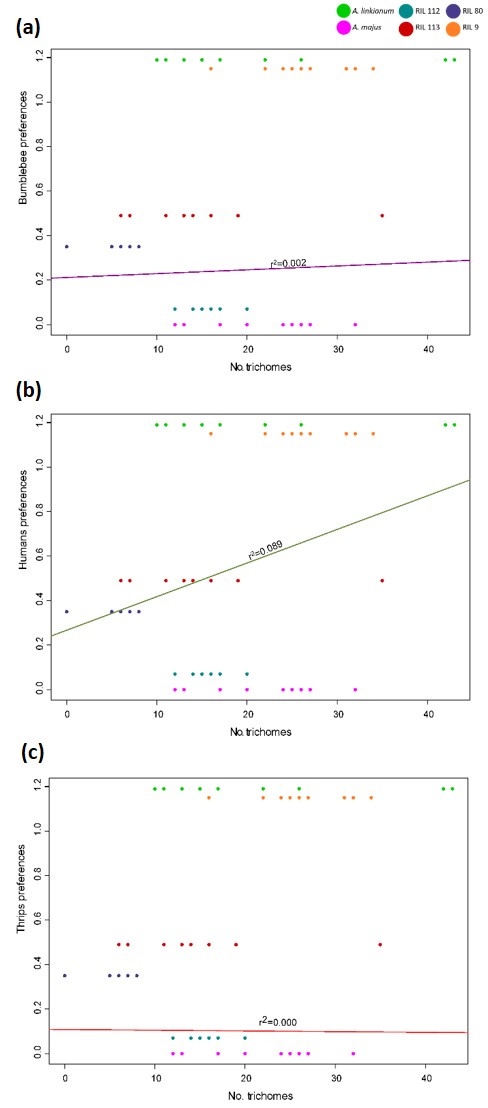


**Supplementary Figure 12.** Correlations between the number of trichomes and the preferences of bumblebees (a), humans (b) and thrips (c).

# Supplementary Tables

**Supplementary Table 1** Volatile compounds found in *A. majus*, *A. linkianum* and RILs 112, 113, 80 and 9. CAS numbers, retention times (RT), linear retention indexes (LRI) calculated and as found in bibliography, differences between calculated and referenced LRIs. Analysed in column HP5 MSVi

| Compound | CAS No | RT | LRI calculated | LRI bibliography | Difference  LRIs (%) | Reference |
| --- | --- | --- | --- | --- | --- | --- |
| Methyl 2-methyl butyrate | 868-57-5 | 2.101 | 779 | 774 | -0.66 | (Triskelion, 2018) |
| β-pinene | 18172-67-3 | 5.000 | 960 | 981 | 2.13 | (Acree & Arn) |
| Sabinene | 3387-41-5 | 5.034 | 961 | 976 | 1.09 | (Acree & Arn) |
| β-myrcene | 123-35-3 | 5.473 | 982 | 991 | 0.92 | (Adams, 1995) |
| (E)-β-ocimene | 3779-61-1 | 6.773 | 1042 | 1038 | -0.43 | (Acree & Arn) |
| Acetophenone | 98-86-2 | 7.139 | 1060 | 1065 | 0.51 | (Adams, 1995) |
| Methyl benzoate | 93-58-3 | 7.763 | 1089 | 1091 | 0.20 | (Adams, 1995) |
| Linalool | 78-70-6 | 7.844 | 1093 | 1098 | 0.49 | (Adams, 1995) |
| Nonanal | 124-19-6 | 7.954 | 1098 | 1104 | 0.57 | (Su Rae Lee, Carlos Macku, 1991) |
| o-acetylphenol | 118-93-4 | 9.039 | 1156 | 1160 | 0.32 | (Triskelion, 2018) |
| Ethyl benzoate | 93-89-0 | 9.196 | 1165 | 1185 | 1.70 | (Acree & Arn) |
| Methyl salicylate | 119-36-8 | 9.597 | 1187 | 1190 | 0.29 | (Adams, 1995) |
| Decanal | 112-31-2 | 9.792 | 1197 | 1204 | 0.57 | (Heinen, 1990) |
| Benzenepropanol | 122-97-4 | 10.235 | 1224 | 1221 | -0.26 | (Kim *et al.*, 2016) |
| 3,5-dimethoxytoluene | 4179-19-5 | 10.815 | 1260 | 1260 | 0.00 | (Kim *et al.*, 2016) |
| (E)-cinnamaldehyde | 14371-10-9 | 10.887 | 1264 | 1283 | 1.45 | (Acree & Arn) |
| Methyl hydrocinnamate | 103-25-3 | 10.959 | 1269 | 1270 | 0.09 | (Lima Neto *et al.*, 2017) |
| o-acetanisole | 579-74-8 | 11.235 | 1286 | 1285 | -0.07 | (Kim *et al.*, 2016) |
| Cinnamyl alcohol | 104-54-1 | 11.320 | 1291 | 1312 | 1.59 | (Acree & Arn) |
| Methyl cinnamate | 103-26-4 | 12.525 | 1372 | 1379 | 0.50 | (Acree & Arn) |
| Eremophilene | 10219-75-7 | 14.068 | 1483 | 1490 | 0.44 | (Acree & Arn) |
| α-farnesene | 502-61-4 | 14.273 | 1499 | 1500 | 0.09 | (Acree & Arn) |
| Hexahydrofarnesyl acetone | 502-69-2 | 18.254 | 1835 | 1770 | -3.69 | (Acree & Arn) |

*References:

**Acree T., Arn H**. Flavornet.

**Adams RP**. **1995**. *Identification of Essential Oil Components by Gas Chromatography/ Mass Spectroscopy*. Allured Publishing Corporation.

**Heinen P**. **1990**. LRI-values and odours of authentic compounds on DB5 column (GC). *University of Reading, Department of Food Science and Technology*.

**Kim S, Thiessen PA, Bolton EE, Chen J, Fu G, Gindulyte A, Han L, He J, He S, Shoemaker BA, *et al.*** **2016**. PubChem Substance and Compound databases. *Nucleic acids research* **44**: D1202-13.

**Lima Neto J de S, Lopes JAD, Moita Neto JM, Lima SG de, Luz CFP da, Citó AM das GL**. **2017**. Volatile compounds and palynological analysis from pollen pots of stingless bees from the mid-north region of Brazil. *Brazilian Journal of Pharmaceutical Sciences* **53**.

**Su Rae Lee, Carlos Macku TS**. **1991**. Isolation and identification of headspace volatiles formed in heated butter. *J. Agric. Food Chem.* **39**: 1972–1975.

**Triskelion**. **2018**. Volatile Compounds in Food. *16.5*.

**Supplementary Table 2** Effect sizes, as the log response ratio, of preferences of bumblebees, thrips and humans for flowers of *Antirrhinum*. Average responses of organisms towards pairs of flowers tested (*A. majus* vs. X). X̅_E_ is the mean response of the organism to *A. linkianum*, RIL 112, RIL 113, RIL 9 or RIL 80 and X̅_C_ is the mean response to *A. majus.*

| Organism | Experiment | Pair 1 | Mean 1 (Xc) | | SD 1 | Pair 2 | Mean 2 (Xe) | SD 2 | N | Xe/Xc | L_index | Rank |
| --- | --- | --- | --- | --- | --- | --- | --- | --- | --- | --- | --- | --- |
| Bumblebeess | Flowers | A_majus | | 4.3 | 1.88 | A_majus | 4.3 | 1.88 | 50 | 1 | 0 | 4 |
|  | Flowers | A_majus | 3.4 | | 1.96 | A_linkianum | 6.8 | 2.1 | 10 | 2 | 0.69 | 2 |
|  | Flowers | A_majus | 3 | | 1.33 | RIL_112 | 7 | 1.33 | 10 | 2.33 | 0.85 | 1 |
|  | Flowers | A_majus | 4.6 | | 1.35 | RIL_113 | 5.4 | 1.35 | 10 | 1.17 | 0.16 | 3 |
|  | Flowers | A_majus | 5.2 | | 1.62 | RIL_9 | 4.8 | 1.62 | 10 | 0.92 | -0.08 | 5 |
|  | Flowers | A_majus | 5.3 | | 2.06 | RIL_80 | 4.7 | 2.06 | 10 | 0.89 | -0.12 | 6 |
|  | Scent | A_majus | 5.15 | | 1.35 | A_majus | 5.15 | 1.35 | 0 | 1 | 0 | 4 |
|  | Scent | A_majus | 6.4 | | 1.17 | A_linkianum | 3.6 | 1.17 | 10 | 0.56 | -0.58 | 6 |
|  | Scent | A_majus | 4.87 | | 1.19 | RIL_112 | 5.13 | 1.19 | 15 | 1.05 | 0.05 | 2 |
|  | Scent | A_majus | 4.6 | | 0.84 | RIL_113 | 5.4 | 0.84 | 10 | 1.17 | 0.16 | 1 |
|  | Scent | A_majus | 5.1 | | 1.79 | RIL_9 | 4.9 | 1.79 | 10 | 0.96 | -0.04 | 5 |
|  | Scent | A_majus | 4.9 | | 1.1 | RIL_80 | 5.1 | 1.1 | 10 | 1.04 | 0.04 | 3 |
| Thrips | Flowers | A_majus | 13.12 | | 3.29 | A_majus | 13.12 | 3.29 | 446 | 1 | 0 | 4 |
|  | Flowers | A_majus | 13 | | 4 | A_linkianum | 15.43 | 4.28 | 199 | 1.19 | 0.17 | 3 |
|  | Flowers | A_majus | 11.78 | | 2.68 | RIL_112 | 16.56 | 3.36 | 255 | 1.41 | 0.34 | 2 |
|  | Flowers | A_majus | 11 | | 1.55 | RIL_113 | 17.83 | 2.79 | 173 | 1.62 | 0.48 | 1 |
|  | Flowers | A_majus | 14.67 | | 2.66 | RIL_9 | 13.17 | 4.07 | 167 | 0.9 | -0.11 | 5 |
|  | Flowers | A_majus | 15.83 | | 3.31 | RIL_80 | 12.83 | 4.75 | 172 | 0.81 | -0.21 | 6 |
|  | Stamens | A_majus | 8.17 | | 3.23 | A_majus | 8.17 | 3.23 | 188 | 1 | 0 | 6 |
|  | Stamens | A_majus | 7.5 | | 1.91 | A_linkianum | 11.75 | 1.5 | 77 | 1.57 | 0.45 | 3 |
|  | Stamens | A_majus | 10.67 | | 3.14 | RIL_112 | 10.83 | 1.17 | 129 | 1.02 | 0.02 | 5 |
|  | Stamens | A_majus | 9.75 | | 3.59 | RIL_113 | 12.5 | 1.91 | 89 | 1.28 | 0.25 | 4 |
|  | Stamens | A_majus | 6.25 | | 1.26 | RIL_9 | 12.75 | 1.26 | 76 | 2.04 | 0.71 | 2 |
|  | Stamens | A_majus | 6 | | 3.08 | RIL_80 | 13.6 | 2.61 | 98 | 2.27 | 0.82 | 1 |
| Humans | Flowers | A_majus | 0.35 | | 0.48 | A_majus | 0.35 | 0.48 | 146 | 1 | 0 | 6 |
|  | Flowers | A_majus | 0.23 | | 0.43 | A_linkianum | 0.77 | 0.43 | 30 | 3.29 | 1.19 | 1 |
|  | Flowers | A_majus | 0.48 | | 0.51 | RIL_112 | 0.52 | 0.51 | 29 | 1.07 | 0.07 | 5 |
|  | Flowers | A_majus | 0.38 | | 0.49 | RIL_113 | 0.62 | 0.49 | 29 | 1.64 | 0.49 | 3 |
|  | Flowers | A_majus | 0.24 | | 0.44 | RIL_9 | 0.76 | 0.44 | 29 | 3.14 | 1.15 | 2 |
|  | Flowers | A_majus | 0.41 | | 0.5 | RIL_80 | 0.59 | 0.5 | 29 | 1.42 | 0.35 | 4 |
|  | Scent | A_majus | 0.63 | | 0.49 | A_majus | 0.63 | 0.49 | 147 | 1 | 0 | 2 |
|  | Scent | A_majus | 0.53 | | 0.51 | A_linkianum | 0.47 | 0.51 | 30 | 0.88 | -0.13 | 3 |
|  | Scent | A_majus | 0.87 | | 0.35 | RIL_112 | 0.13 | 0.35 | 30 | 0.15 | -1.87 | 6 |
|  | Scent | A_majus | 0.57 | | 0.5 | RIL_113 | 0.43 | 0.5 | 30 | 0.76 | -0.27 | 4 |
|  | Scent | A_majus | 0.41 | | 0.5 | RIL_9 | 0.59 | 0.5 | 29 | 1.42 | 0.35 | 1 |
|  | Scent | A_majus | 0.75 | | 0.44 | RIL_80 | 0.25 | 0.44 | 28 | 0.33 | -1.1 | 5 |
